# Genome-Wide Association Analysis of Mucilage and Hull Content in Flax (*Linum usitatissimum* L.) Seeds

**DOI:** 10.3390/ijms19102870

**Published:** 2018-09-21

**Authors:** Braulio J. Soto-Cerda, Sylvie Cloutier, Rocío Quian, Humberto A. Gajardo, Marcos Olivos, Frank M. You

**Affiliations:** 1 Agriaquaculture Nutritional Genomic Center (CGNA), Las Heras 350, Temuco 4781158, Chile; rocio.quian@cgna.cl (R.Q.); humberto.gajardo@cgna.cl (H.A.G.); marcos.olivos@cgna.cl (M.O.); 2 Ottawa Research and Development Centre, Agriculture and Agri-Food Canada, Ottawa, ON K1A 0C6, Canada; sylvie.j.cloutier@agr.gc.ca (S.C.); frank.you@agr.gc.ca (F.M.Y.); 3 Morden Research and Development Centre, Agriculture and Agri-Food Canada, Morden, MB R6M 1Y5, Canada

**Keywords:** flaxseed, *Linum usitatissimum*, GWAS, seed mucilage content, seed hull content, single nucleotide polymorphism (SNP)

## Abstract

New flaxseed cultivars differing in seed mucilage content (MC) with low hull content (HC) represent an attractive option to simultaneously target the food and feed markets. Here, a genome-wide association study (GWAS) was conducted for MC and HC in 200 diverse flaxseed accessions genotyped with 1.7 million single nucleotide polymorphism (SNP) markers. The data obtained for MC and HC indicated a broad phenotypic variation and high (~70%) and a moderate (~49%) narrow sense heritability, respectively. MC and HC did not differ statistically between fiber and oil morphotypes, but yellow-seeded accessions had 2.7% less HC than brown-seeded ones. The genome-wide linkage disequilibrium (LD) decayed to *r*^2^ = 0.1 at a physical distance of ~100 kb. Seven and four quantitative trait loci (QTL) were identified for MC and HC, respectively. Promising candidate genes identified include *Linum usitatissimum* orthologs of the *Arabidopsis thaliana* genes *TRANSPARENT TESTA 8*, *SUBTILISIN-LIKE SERINE PROTEASE*, *GALACTUROSYL TRANSFERASE-LIKE 5*, *MUCILAGE-MODIFIED 4*, *AGAMOUS-LIKE MADS-BOX PROTEIN AGL62*, *GLYCOSYL HYDROLASE FAMILY 17*, and *UDP-GLUCOSE FLAVONOL 3-O-GLUCOSYLTRANSFERASE*. These genes have been shown to play a role in mucilage synthesis and release, seed coat development and anthocyanin biosynthesis in *A. thaliana*. The favorable alleles will be useful in flaxseed breeding towards the goal of achieving the ideal MC and HC composition for food and feed by genomic-based breeding.

## 1. Introduction

Flaxseed (*Linum usitatissimum* L.), one of the oldest crops, has been used as human food and animal feed since ancient times [1]. The two main morphotypes of cultivated *L. usitatissimum* are oil morphotype (flaxseed) and fiber morphotype (fiber flax). Flaxseed plants are shorter, more branched, and larger seeded, and branches cover a greater proportion of the main stem compared to fiber flax. Flaxseed currently enjoys new prospects in the functional food market because of growing consumer interest in food with health benefits [1]. Flaxseed is rich in bioactive compounds, such as α-linolenic acid (omega-3) that have cardiovascular benefits, lignans with anticancer properties, and insoluble and soluble fiber (mucilage) that is capable of lowering cholesterol and insulin [2].

Flaxseed mucilage is a heterogeneous polysaccharide composed of xylose, arabinose, glucose, galactose, rhamnose, and fructose [3] that can be purified into neutral and acidic polymers. Mucilage abounds in the seed coat, where it makes up to 8–10% of the seed weight [4]. Mucilage synthesis is tightly linked to seed coat development [5] and both tissues form the seed hull, a structure representing 37–48% of the seed weight [6,7]. These two fractions, rich in polysaccharides, are components of the flaxseed meal, primarily used as a protein rich livestock and poultry feed [6,8]. Absorption of flaxseed meal′s advantageous 31–45% protein content [9] may be hindered by mucilage and cell wall polysaccharides. This is due to the swelling capacity of polysaccharides in the digestive tract of monogastric animals that causes concomitant growth depression and reduced feed efficiency [7,10]. In that context, reduction of mucilage (MC) and hull (HC) contents in flaxseed meal is desirable to achieve improved feeding value for livestock and poultry. Studies of flaxseed mucilage degradation are focused on chemical retting, enzyme retting, and steam explosion [11]. Reduction of the hull content in flaxseed and rapeseed meal has been achieved through dehulling methods [12] and the use of yellow-seeded genotypes [7,13]. Food and feed markets demand flaxseed cultivars differing in mucilage and hull content. It is, therefore, crucial to decipher the genetic factors underlying these complex traits in order to accelerate the development of market-specific flaxseed cultivars.

In the model plant *Arabidopsis thaliana*, the genes necessary for the synthesis, modification, and release of mucilage, as well as seed coat development, are well understood [5,14]. Putative flax orthologs of the *RHAMNOSE SYNTHASE* (*AtRHM1*), *GALACTURONOSYLTRANSFERASE-LIKE 3* (*GATL3*), *GALACTURONOSYLTRANSFERASE 11* (*GAUT11*), *XYLOGLUCAN ENDOTRANSGLUCOSYLASE/HYDROLASE 3*(*XTH3*), and *ALPHA-XYLOSIDASE-1* (*AtBXL1*), involved in mucilage production, have been identified using gene expression analysis during seed development [15]. Similarly, putative flax orthologs of the *TRANSPARENT TESTA 3*, *4*, *5*, and *7* (*TT3*, *TT4*, *TT5*, and *TT7*), *FLAVONOL SYNTHASE* (*FLS*) and *BANYULS* (*BAN*), involved in flavonoids synthesis during seed coat development, have also been identified [15].

Genetic variation for MC and HC in flaxseed has been assessed [4,7,16,17,18] but no quantitative trait loci (QTL) have been reported so far. QTL for *Fusarium* wilt resistance [19], powdery mildew [20], iodine value, palmitic, linoleic, and linolenic acids [21,22], and seed and flower color [23], were reported. QTL for seed protein, cell wall, straw weight, yield-related traits, and phenological traits, have also been reported using bi-parental mapping and association mapping [22,24,25]. Recently, genome-wide association studies (GWAS) have been conducted for agronomic and seed quality traits using thousands of single nucleotide polymorphism (SNP) loci [26,27]. GWAS mines the natural sequence diversity within a species and captures historical recombination events. It is therefore a suitable approach to discover loci that control complex traits, leading to a higher mapping resolution to facilitate the identification of candidate genes [28]. Thus, the suite of genomic tools available for flaxseed genetic studies [21,22,26,27,29,30,31,32] make genomic evaluation of MC and HC feasible.

The objective of this research was to identify QTL and candidate genes contributing to mucilage content and hull content that could be capitalized upon to assist in breeding flaxseed cultivars with different mucilage content and with reduced hull content. Improving these traits will increase seed value of this important cash crop.

## 2. Results

### 2.1. Phenotypic Evaluation

**Figure 1:**
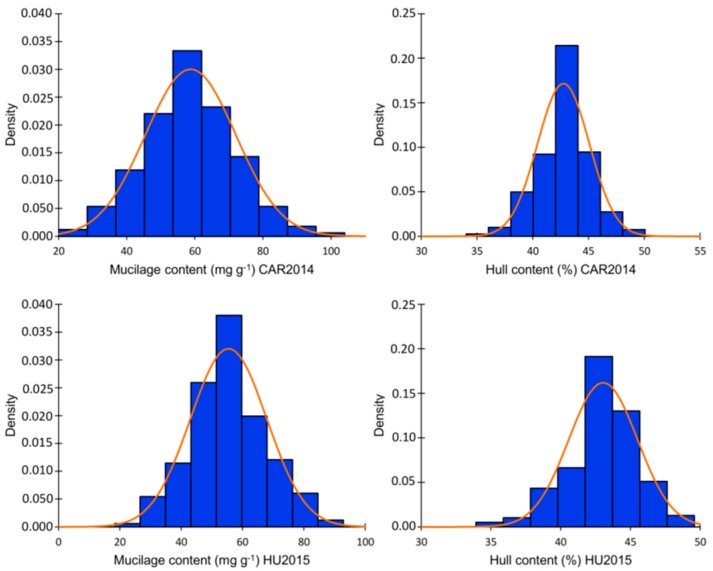
Mucilage (MC) and hull (HC) contents distribution in the association panel in two environments: CAR2014 = Vilcún location 2014, HU2015 = Huichahue location 2015. Values represent the mean of three biological replicates for each trait.

Two hundred flax accessions from the Canadian flax core collection were planted in two environments. Evaluation of MC and HC showed a normal distribution across the two environments according to the Shapiro–Wilk normality test and normality plots (Table S1, Figure S1). Variance component analysis indicated significant effects of genotype, environment, and genotype × environment interaction, according to the Wald statistic (*p* < 0.001). The phenotypic variation for MC in Vilcún location 2014 (CAR2014) ranged from 23.52 to 103.57 mg g^−1^ with an average of 58.67 mg g^−1^. A lower variation was observed for MC in Huichahue location 2015 (HU2015), which ranged from 18.88 to 91.90 mg g^−1^ with an average of 55.04. mg g^−1^ HC variation ranged from 35.56 to 48.59% in CAR2014 and from 35.73 to 48.59% in HU2015 (Figure 1, Table S1). MC and HC were significantly positively correlated in CAR2014 and HU2015 with coefficients of 0.28 and 0.29, respectively. Narrow sense heritability (*h*^2^) for MC attained 70.7 and 73.8% in CAR2014 and HU2015, respectively. Lower *h*^2^ of 51.4 and 46.2% for HC at CAR2014 and HU2015 were observed. MC did not differ statistically between flax morphotypes nor seed color classes, according to the Kruskal–Wallis non-parametric test. The average MC was 55.33 and 56.63 mg g^−1^ for the fiber and oil morphotypes, respectively (*p* = 0.651) (Figure S2a). The average MC registered values of 56.63 and 59.22 (*p* = 0.517) for the brown and yellow seeded classes, correspondingly. The average HC did not differ statistically between flax morphotypes (fiber = 43.41%, oil = 42.79%; *p* = 0.373). On the other hand, yellow-seeded genotypes averaged 2.66% less HC than brown seeded accessions (*p* = 3.2 × 10^−5^) (Figure S2b).

### 2.2. Population Structure and Linkage Disequilibrium

**Figure 2:**
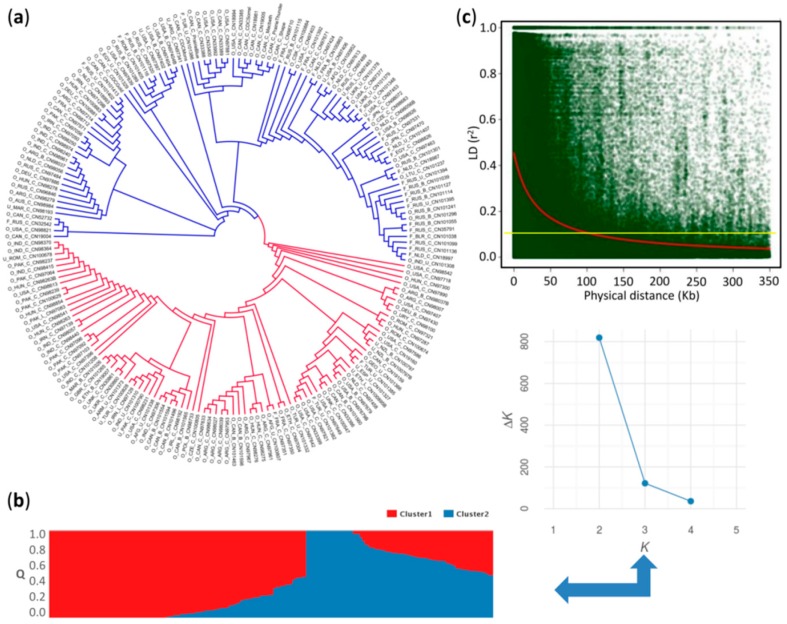
Population structure and genome-wide linkage disequilibrium decay. (**a**) Neighbor-joining (NJ) tree for 200 flax accessions based on 779,914 single nucleotide polymorphisms (SNPs); (**b**) Model-based population structure of 200 flax accessions belonging to two clusters predefined by the STRUCTURE software. Each accession is represented by a vertical bar. The color subsections within each vertical bar indicate membership coefficient (Q) to different clusters; (**c**) Genome-wide linkage disequilibrium decay of *r*^2^ values (red line), against physical distance (kb) using the Hill and Weir (1988) function in *L. usitatissimum*. Yellow line indicates the cutoff value (*r*^2^ = 0.1) used to determine the genome-wide linkage disequilibrium (LD) block size.

The dendrogram based on 771,914 SNPs and the STRUCTURE plot grouped the 200 individuals into two major clusters arbitrarily assigned as “red” and “blue” (Figure 2a,b). In the *K* against Δ*K* plot, a break in the slope was clearly observed at *K* = 2 (Figure 2b). The red cluster comprised almost exclusively genotypes belonging to the oil morphotype, while the blue cluster included both flax morphotypes. The coefficient of population differentiation (*F_ST_* = 0.08) indicated a weak population structure between the two clusters.

**Figure 3:**
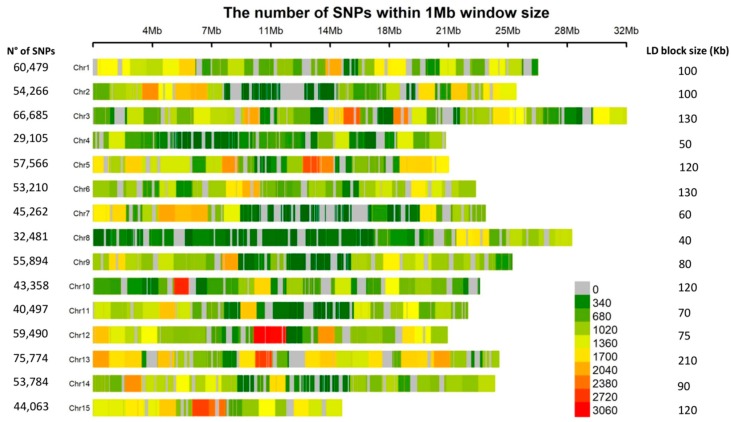
Single nucleotide polymorphism (SNP) density plot across the *L. usitatissimum* genome. Numbers of SNPs and LD blocks are also indicated for each of the 15 chromosomes.

The genome-wide linkage disequilibrium (LD) decayed to *r*^2^ = 0.1 at a physical distance of ~100 kb (Figure 2c). Intrachromosomal LD decayed to *r*^2^ = 0.1 at a distance between marker pairs ranging from ~40 kb on chromosome 8 to ~210 kb on chromosome 13 (Figure S3). A highly significant positive correlation (*r* = 0.75, *p* = 0.0012) between marker density and the intrachromosomal LD blocks was observed (Figure 3). For example, chromosomes 4 and 8 with the smallest number of markers, and chromosomes 6 and 13 with the largest, displayed the fastest and slowest LD decays, respectively (Figure 3 and Figure S3). The fast LD decays observed in this association panel are indicative of its advantageous potential for reducing QTL intervals and fine mapping of candidate genes for MC and HC.

### 2.3. Genome-Wide Association Analysis

Three GWA models were tested, including GLM-Q, GLM-PCA, and MLM-K. According to the quantile-qualtile (Q–Q) plot results, the GLM-Q model showed a strong skew toward significance for every trait (Figure S4a), indicating that the Q matrix was insufficient to account for population structure and cryptic relatedness. Conversely, the MLM-K, which only used the kinship matrix, led to an overcorrection of these confounding factors, particularly for HC (Figure S4b). The GLM-PCA was tested with 5 and 10 PCA covariates for HC and MC, accounting for 30.1 and 37.1% of the variation, respectively. Both GLM-5PCA and GLM-10PCA models performed well in controlling the rate of false positives, providing suitable statistical power to identify significant marker–trait associations for MC and HC (Figure S5). Therefore, the GLM-PCA model was applied for GWA in this study.

GWA analysis identified 12 and 17 significant associations for MC in CAR2014 and HU2015, respectively (*p* < −log_10_ (*P*) = 6.88), and markers Lu5-3808878, Lu7-13225294, and Lu11-2498303 were significant in both environments (Table 1, Figure S5). Various significant SNP markers fell into the same LD blocks. For example, five other significant markers surrounded the peak SNP Lu5-3808878 (Figure S5), thus, they were considered the same QTL. Following this criterion, seven QTL were delineated on chromosomes 2, 3, 5, 7, and 11. The peak SNPs of these QTL accounted for 11.8 to 17.3% of phenotypic variation, and the combined three consistent QTL accounted for 43.6% of the MC variation (Table 1).

A total of three and four significant associations were detected for HC in CAR2014 and HU2015, respectively (*p* < −log_10_ (*P*) = 6.88). Markers Lu7-6577527 and Lu13-2803224 were significant in both environments (Table 1). The four QTL identified on chromosomes 7, 10, 12, and 13 explained between 13.8% and 17.8% of the HC variation. The two consistent QTL Lu7-6577527 and Lu13-2803224, accounted for a combined 33% of the HC variation (Table 1).

**Figure 4:**
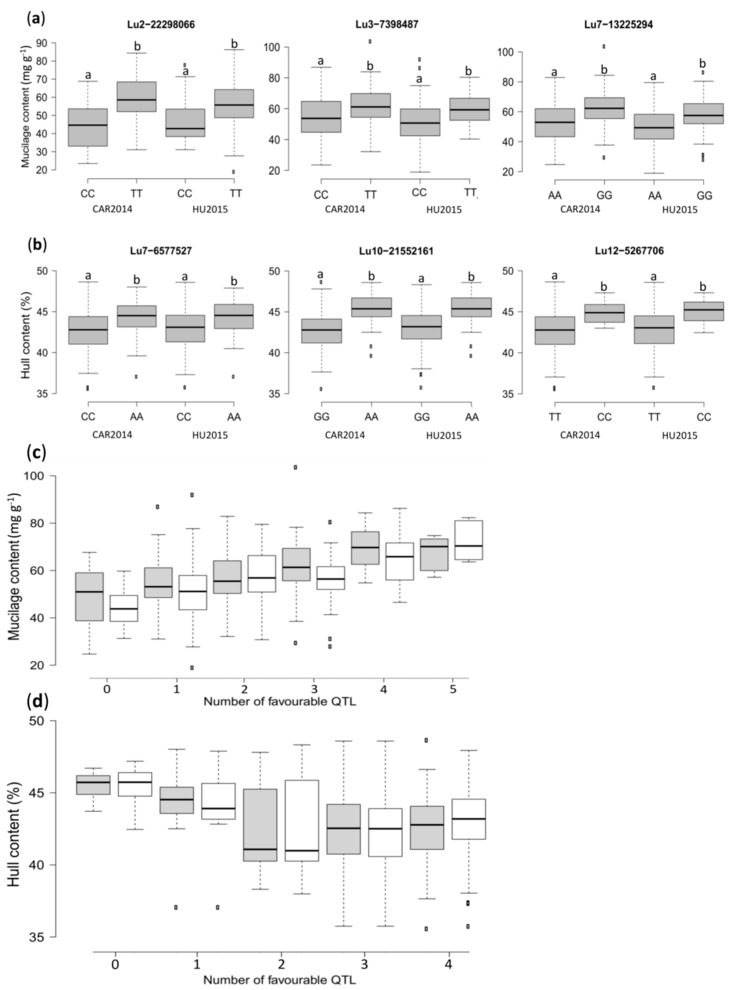
Box plots illustrating the phenotypic differences between flaxseed accessions carrying different alleles of the significant SNPs, and combined phenotypic effects of favorable QTL in the association panel. (**a**) Mucilage content (MC); (**b**) Hull content (HC). CAR2014 = Vilcún location 2014, HU2015 = Huichahue location 2015. Different letters indicate significant statistical differences according to the Kruskal-Wallis non-parametric test (*p* < 0.05); (**c**) QTL effect for MC; (**d**) QTL effect for HC. Grey and white boxplots represent the CAR2014 and HU2015 locations, respectively.

The peak SNPs effect for MC and HC were all significant according to the non-parametric Kruskal–Wallis test (*p* < 0.05), except for Lu3-26033342 associated with MC (Figure 4a,b and Figure S6). Accessions with a thymine (T) allele at Lu2-22298066 displayed, on average, an increase of 15.3 and 9.4 mg g^−1^ in MC, compared to accessions with a cytosine (C) allele in CAR2014 and HU2015, respectively (Figure 4a). Similarly, accessions with a “T” allele at Lu3-7398487 had, on average, 6.64 and 8.4 mg g^−1^ higher MC compared to accessions with a “C” allele in CAR2014 and HU2015, correspondingly.

Accessions with a guanine (G) allele at Lu7-13225294 had, on average, 8.56 and 7.71 mg g^−1^ more mucilage compared to accessions carrying an adenine (A) allele in CAR2014 and HU2015, respectively (Figure 4a). The allelic effect for the other four peak SNPs is illustrated in Figure S6. The allelic effect of peak SNPs for HC revealed that accessions harboring a “C” allele at Lu7-6577527 had, on average, 1.4 and 1.3% less HC compared with “A” allele genotypes in CAR2014 and HU2015, correspondingly (Figure 4b). On average, HC was reduced by 1.4 and 1.3% (Lu7-6577527) to 2.6 and 2.7% (Lu13-2803224) in CAR2014 and HU2015, respectively (Figure 4b and Figure S6).

The combined QTL effect revealed that the MC of accessions harboring none of the favorable QTL alleles averaged 44.6 and 48.9 mg g^−1^, compared to 72.1 and 67.6 mg g^−1^, for those with five favorable alleles in CAR2014 and HU2015, respectively (Figure 4c). No accession had all seven favorable QTL alleles. The combined QTL effect for HC indicated that genotypes with none of the favorable QTL alleles averaged 45.5% and 45.3% HC compared to genotypes with four favorable QTL alleles, in which HC averaged 42.7% and 42.9% in CAR2014 and HU2015, respectively (Figure 4d).

### 2.4. Identification of Candidate Genes

The LD blocks harboring the peak SNPs were mined for genes relevant to MC and HC using the *L. usitatissimum* v.1.0 reference genome. A total of 204 and 118 candidate genes were identified for MC and HC, respectively (Table 2 and Table S2). Several genes ascribed to carbohydrate metabolism, seed mucilage synthesis, modification, and release, and cell wall synthesis and modification were identified at the MC QTL loci (Table 2). Five particularly promising candidate genes were identified. The SNP marker Lu3-26033342 was located 58.92 and 49.60 kb from Lus1007101 and Lus10007083 that encode the ortholog of *A. thaliana*’s *TRANSPARENT TESTA 8* (*TT8*) and *SUBTILISIN-LIKE SERINE PROTEASE* (*SBT1.7*) (Figure S5a, Table 2). In another independent QTL on chromosome 3, the SNP marker Lu3-25559600 was located 64.41 and 67.02 kb from Lus10009311 and Lus10009288 that encode the ortholog of *A. thaliana*’s *GALACTUROSYL TRANSFERASE-LIKE 5* (*GATL5*) and *MUCILAGE-MODIFIED 4* (*MUM4*). On chromosome 5, Lu5-3508878 is located 100.78 kb from Lus10008285, an ortholog of another *A. thaliana* gene implicated in mucilage transcriptional regulation, *NAC-REGULATED SEED MORPHOLOGY 1* (*NARS1*) (Figure S5a, Table 2).

Genes related to embryo, endosperm, and seed coat development, cell wall biogenesis/degradation, anthocyanin biosynthesis, and seed dormancy, were found at QTL loci associated with HC (Table 2 and Table S2). Among the relevant candidate genes, Lus10035456 encodes the ortholog of *A. thaliana*’s *AGAMOUS-LIKE MADS-BOX PROTEIN AGL62* (*AGL62*) and is located 11.40 kb from the SNP marker Lu7-6577527 (Figure S5b, Table 2). On chromosome 12, Lu12-5267706 was situated 39.93 kb from Lus10018306 that encodes the ortholog of *A. thaliana*’s *GLYCOSYL HYDROLASE FAMILY 17* (*GH17*). Two other interesting candidate genes, Lus10026902 and Lus10026926, were situated 96.87 and 238.19 k, respectively, from the SNP marker Lu13-2803224. Lus10026902 and Lus10026926 encode the ortholog of *A. thaliana*’s *LARIAT DEBRANCHING ENZYME* (*DBR1*) and *UDP-GLUCOSE FLAVONOL 3-O-GLUCOSYLTRANSFERASE* (*UGT79B1*), respectively.

## 3. Discussion

### 3.1. Phenotypic Variation of Mucilage and Hull Contents

Flaxseed mucilage and seed hull possess valuable nutritional and rheological attributes [33,34] but are also known to affect animal performance [7]. The presence of mucilage and fiber components (i.e., acid detergent lignin) in flaxseed meal reduces the energy uptake in both monogastric and ruminant animals [35]. Therefore, knowledge about the phenotypic variation and genetic control of seed mucilage content (MC) and hull content (HC) is pivotal to better design breeding strategies aiming to improve the overall food and feed value of flaxseed. The broad phenotypic variation of MC and HC in the association panel and the degree of additivity of the genetic components hint at the potential for improving flaxseed for either high or low MC and reduced HC through marker-assisted selection.

Kaewmanne et al. [4] reported MC ranging from 1.8 to 2.9% in seven Italian flaxseed cultivars, while Oomah et al. [16] found that MC ranged from 3.6 to 8.0% in 109 flaxseed accessions. We found a slightly wider range from 2 to 10% in our diversity panel. Little information exists for HC variation in large collections of flaxseed. In general, HC ranges from 22–27% to 36–48% were reported in mechanically treated and hand-dissected seeds, respectively [7,36], which is much higher than canola at 18.6% and soybean at 16.1% [6]. Reduction of HC can be achieved through the use of yellow-seeded cultivars, known to contain higher oil content and less HC than their brown-seeded counterparts [7,37]. Indeed, the yellow-seeded accessions displayed a lower HC compared to the brown-seeded genotypes. Nevertheless, caution should be exercised in adopting yellow-seeded flaxseed cultivars for reduced HC flaxseed because their susceptibility to natural splitting and mechanical cracking of the seed coat can negatively affect seed quality [38]. Consequently, breeding and seed tests to mechanical damage during harvesting should be conducted together in order to identify the ideal HC that would ensure seed mechanical resistance. All considered, our association panel harbored abundant phenotypic variation for dissecting the genetic landscape of MC and HC.

### 3.2. Population Structure and Linkage Disequilibrium

When the main factors accounting for population subdivision correlate with a trait under study (i.e., geographic distribution and flowering time), then marker–trait associations will undergo a more accentuated inflation of observed *p*-values as effect of the structure confounding factor [39]. In flaxseed, population structure has been assessed in varying numbers of accessions, where geographic origin and flax morphotype seemed to have been the main factors underlying population subdivisions [40,41,42]. In our association panel, the “red” and “blue” clusters were slightly differentiated (*F_ST_* = 0.08), with a weak morphotypic effect on dendrogram topology, possibly due to the small number of fiber types (*n* = 33) compared to the larger number of oilseed type accessions (*n* = 153).

Linkage disequilibrium (LD) is the main factor influencing marker density requirement and mapping resolution in GWAS. Mating system and genetic diversity influence LD decay. LD decays more rapidly in outcrossing plant species than in self-pollinated plants [43] and, similarly, in wild relatives and landraces compared to modern cultivars [44]. Here, we observed a rapid LD decay for most of the chromosomes, comparable to some maize commercial elite inbred lines [45] and faster than winter-type *Brassica napus* (480 to 1283 kb, *r*^2^ = 0.1) [46]. Therefore, the 200 flaxseed accessions of our diversity panel are expected to contain plentiful allelic diversity, as suggested by the generally short LD blocks for the 15 chromosomes, thereby assisting the search for candidate genes through efficient narrowing of the putative QTL regions.

### 3.3. Genome-Wide Association Analysis

Several general (GLM) and mixed (MLM) linear models have been proposed to control both population structure and cryptic relatedness [47,48,49]. In flax, MLM has been the preferred association model for multiple traits [24,25,26,42]. The “red” and “blue” clusters were weakly differentiated, and MC and HC between flax morphotypes was not statistically significant (Figure S2a,b), in contrast to a report comparing *indica* and *japonica* rice types assessed for 34 traits [39]. Hence, the genetic architecture of MC and HC seem to be only weakly correlated with population and family structures, and GLM-PCA was sufficient to control the rate of false positive associations.

The discovery of QTL for agronomic and economically important traits in crops is of great importance for marker-assisted breeding. This is the first report of QTL for MC in flax, likely because this trait has not been a breeding priority in the most important breeding programs of the world [18]. In the present study, GWAS identified seven QTL for MC, and their effects clearly suggest the promise of marker-assisted selection for modifying MC.

Chromosome 3′s multiple MC QTL harbored candidate genes orthologous to Arabidopsis *TT8* gene, which is part of a transcription factor complex that, along with *GLABRA2* (*GL2*), regulates *MUM4* gene expression [50]. *MUM4* is required to produce rhamnose, a key substrate for mucilage biosynthesis [50], and chromosome 3 Lus10009311 is its flax ortholog. In Arabidopsis, *GATL5* encodes a glycosyltransferase involved in rhamnogalacturonan I (RG I) backbone synthesis [51]. The presence of a *L. usitatissimum* ortholog Lus10009311 in a LD block, with a peak SNP for MC, corresponds to the expected role of RG I synthesis. Arabidopsis gene *SBT1.7* triggers the activation of cell wall-modifying enzymes necessary for mucilage release upon imbibition [52]. In line with the expected seed coat mucilage dynamics, we identified two orthologous copies of this gene in two independent QTL (Table 2). Arabidopsis *PECTIN METHYLESTERASE INHIBITOR 6* (*PMEI6*) mutants were defective in seed coat mucilage release [53]. An ortholog of the Arabidopsis gene, *PECTIN METHYLESTERASE 36* (*PME36*), another family member, was located at one of the MC QTL loci identified herein. While *PME36* has not been shown to be involved in mucilage release, it might participate indirectly because it exerts a similar role to that of *PMEI6* in pectin synthesis and cell wall modification [54].

Oil content is an economically important but genetically complex trait. MC is negatively correlated with oil content, therefore, reducing MC should facilitate increasing oil content. Indeed, reduced accumulation of mucilage accompanied by increased oil content was observed in Arabidopsis *MUM4* or *GL2* mutants [55]. We observed a significant negative correlation (*r* = −0.15, *p* = 0.03) between MC and oil content in the association panel (data not shown). This is perhaps due to increased carbon allocation to the embryo in reduced or no seed coat mucilage synthesis in low MC accessions as proposed in Arabidopsis [55].

Increasing seed oil content and reducing the fiber fraction of the meal have been important goals in oil crop breeding. In *B. napus* and *L. usitatissium*, seed coat thickness or HC are negatively correlated with seed oil and protein content, as well as seed color [56,57,58]. QTL for seed coat color to indirectly increase oil content and minimize HC have been identified in *B. napus* and soybean [37,59,60]. In flax, a pleiotropic QTL controlling yellow seed and white flower color was recently dissected at the molecular level, but its effect on HC has not been addressed [23]. Here, we identified four QTL whose effects reduced HC by 2.6%, on average. Chromosome 7 harbored Lus10035456, which resembles the *A. thaliana* transcription factor *AGL62*. *AGL62* mutants initiated embryo and endosperm formation, but failed to form a seed coat [61]. Light seed color and low HC are thought to coincide because the biochemical pathways leading to lignin and pigment synthesis share common precursors [59]. In Arabidopsis, the core components of seed coat pigments are proanthocyanidins (PAs) [62]. Chromosome 12 encompassed three candidate genes including the ortholog of Arabidopsis *O-GLYCOSYL HYDROLASES FAMILY 17* gene. *GH17* is coexpressed with *TT12*, *AHA10*, and *BAN*, that might process glycosylated flavan-3-ol monomers, leading to accumulation of PAs in the seed coat [63]. In black seed soybean, a *UDP-GLUCOSE:FLAVONOID 3-O-GLUCOSYLTRANSFERASE* (*UGT78K1*), was isolated from the seed coat, a key enzyme that catalyzes the final step in anthocyanin biosynthesis [64]. Chromosome 13 contained Lus10026926, an ortholog of the *A. thaliana UGT79B1*, a gene also involved in anthocyanin biosynthesis. Yellow seed color stems from the blocked biosynthesis of PAs that impart the brown color to the seed coat [65]. The flaxseed meal derived from brown-seeded cultivars contains PAs that negatively affect protein digestion [66], hence low PA meal is preferred in animal ration. Additional advantages of modifying the seed color and reducing MC and HC include higher limpidity of the crude oil from the removal of gum-like residues and dark pigments, higher protein content and better feeding value of flaxseed meal for livestock and poultry [7].

Few accessions combined favorable alleles for reduced MC and HC. It should be possible to combine these attributes in a single genotype through the pyramiding of the respective favorable alleles owing to the fact that the significant QTL for both traits did not co-locate in the flax genome. The development of yellow-seeded cultivars with low HC and either low or high MC for different industrial uses is an opportunity to increase market share and value.

## 4. Materials and Methods

### 4.1. Plant Material, Field Trials, Phenotyping, and Statistical Analyses

A total of 200 *L. usitatissimum* accessions from the Canadian flax core collection [67] were selected for this study based on their geographic distribution and genetic diversity (Table S3). The 200 genotypes were planted in 2014 and 2015 at the Agriaquaculture Nutritional Genomic Center (CGNA) experimental stations located in Vilcún (CAR2014) and Huichahue (HU2015), La Araucania region, Chile, using a completely randomized design (CRD) with three biological replicates. Genotypes were arranged in rows and columns in order to take into account spatial heterogeneity.

The seed mucilage content (MC) was determined in three biological replicates following the procedure described by Kaewmanee et al. [4] with minor modifications. A total of 2 g of seeds were incubated in 20 mL of water at 100 °C for 15 min in 50 mL Falcon tubes. Next, the tubes were shaken for 30 min at 250 rpm. The soluble extract was recovered by centrifugation at 6132 relative centrifugal force (RCF) for 30 min, and the mucilage fraction was precipitated by incubating in 30 mL of ethanol (95%) overnight at 4 °C. The seeds were recovered, and the extraction procedure was carried out twice more to maximize mucilage recovery. The mucilage pellet was weighed and expressed as milligrams of mucilage per gram of seed (mg g^−1^).

HC was determined in three biological replicates by separating the hull from the embryos using a dissecting needle and tweezers from 50 seeds after imbibition in water for 24 h. Both fractions were dried at 90 °C for 4 h before their dry weights were measured. HC was expressed as (hull dry weight/(hull dry weight + embryo dry weight)) × 100, averaged from 50 seeds.

Variation of phenotypic data was analyzed individually for each environment using a restricted maximum likelihood (REML) analysis. Spatial correction in row and column directions was used with different variance–covariance structures. Spatial models were compared with Akaike information criterion (AIC) and Bayesian information criterion (BIC), and the most appropriate model in each environment was used to obtain a best linear unbiased estimate (BLUEs) for mucilage and hull contents in GenStat v.16 [68]. Descriptive statistics and Shapiro–Wilk normality test were conducted in the R package MVN [69]. Narrow sense heritability (*h*^2^) was estimated using variance components from TASSEL v.5.2.31 [70]. Trait *h*^2^ estimates were computed using the equation: *h*^2^ = σ^2^_a_/σ^2^_a_ + σ^2^_e_, where σ^2^_a_ is the additive genetic variance and σ^2^_e_ is the residual error variance [70].

### 4.2. Whole Genome Resequencing and SNP Calling

Genotyping by sequencing (GBS) methodology was adopted to genotype the 407 accessions from the Canadian flax core collection. The 407 individuals were grown in pots in a greenhouse with a 16 h light and 8 h dark cycle. Young leaf tissues from single plants were collected for DNA extraction using the DNeasy 96 Plant kit (Qiagen, Mississauga, ON, Canada) according to manufacturer’s instructions. Genomic DNA was quantified, sheared, size-selected, and libraries were constructed for each genotype by the Michael Smith Genome Sciences Centre of the BC Cancer Agency, Genome British Columbia (Vancouver, BC, Canada) which also sequenced the libraries generating 100 bp paired-end reads on the Illumina HiSeq 2000 platform (Illumina Inc., San Diego, CA, USA). A total of 26.875 billion 100 bp pair-end reads were generated, corresponding to 6587 MB sequences and 15.5× genome equivalents of the reference genome (~370 MB) [32,71], on average, per individual.

All reads from each individual of the population was aligned to the flax reference sequence (the flax pseudomolecules v2.0) [72] using BWA (v0.6.1) [73] with default parameters. The alignment file for each individual was used as input for SNP discovery using the software package SAMtools [74]. SAMtools converts the sequence alignment files from sequence alignment/map (SAM) to sorted binary alignment/map (BAM) format and call all potential variants (SNP and indels) into the pileup files. Then, all variants were further filtered to get a set of high-quality SNPs. SNPs were filtered by the following criteria: (1) candidate SNP loci must be more than 10 bp away from each other; (2) each candidate SNP must have three mapped reads on the region; and (3) all the singleton SNPs were excluded. All steps of this procedure were implemented in an annotation-based genome-wide SNP discovery (AGSNP) pipeline [75,76]. The coordinates of all SNPs were extracted from the chromosome-based flax pseudomolecules v2.0 [72] totaling 1.7 million SNPs. Two hundred accessions, as previously mentioned, were selected for this study.

### 4.3. Population Structure, LD, Genome-Wide Association Study, and Candidate Genes

Population structure was estimated with 259 neutral SSR loci [41] distributed across flax’s 15 chromosomes. The software STRUCTURE v.2.3.4 [47] was employed with predefined numbers of genetic clustering (K) from 1–5, using 50,000 burn-in iterations, followed by 100,000 MCMC across five independent runs for each K values. The number of clusters (K) was calculated with the Evanno method [77] implemented in the R package POPHELPER v.1.1.10 [78]. A total of 771,914 SNPs, filtered from the 1.7 million SNPs by removing those with a minor allele frequency <0.05 and >10% missing data, were used to produce a dendrogram using the neighbor-joining (NJ) algorithm implemented in TASSEL v.5.2.31 [70]. Genome-wide linkage disequilibrium (LD) and intrachromosomal LD between pairs of SNPs using the 771,914 filtered SNPs were estimated using squared allele frequency correlations (*r*^2^) in TASSEL v.5.2.31 [70]. LD values were plotted against physical distance to determine the LD decay using the Hill and Weir [79] function. A cut-off value of *r*^2^ = 0.1 was set to estimate the average LD blocks [41].

GWAS was performed in TASSEL v.5.2.31 [70] using the 771,914 filtered SNPs. Three models were evaluated, including GLM-Q, GLM-PCA, and MLM-K. The Q matrix generated from STRUCTURE was used as a cofactor to adjust for population stratification (GLM-Q). A GLM-PCA was assessed, including up to ten principal component covariates. The ten PCAs were generated in TASSEL v.5.2.31 [70] with 105,038 SNPs (MAF > 0.05 and at least 95% present among the 200 genotypes). For the MLM-K, a kinship matrix was created in TASSEL v.5.2.31 [70] with the set of 105,038 SNPs, and used as covariate to account for cryptic relatedness. A quantile–quantile (Q–Q) plot was displayed using the R package qqman [80] to evaluate the fitness and efficiency of the different models. The final Manhattan plots were also displayed using the qqman package [80]. The Bonferroni correction (0.1/771,914 = −log (*P*) = 6.88) was used as threshold for the significance of marker–trait associations.

To identify candidate genes associated with significant SNPs, the Jbrowse feature of Phytozome v.12.1 (http://phytozome.jgi.doe.gov/pz/portal.html) was used to examine the *L. usitatissimum* v.1.0 genome [71] for genes relevant to MC and HC in flaxseed. As mentioned above, a cut-off value of *r*^2^ = 0.1 was set to estimate the average LD block for each chromosome. The defined physical distance was used to pinpoint candidate genes on either side of the most significant SNPs. A plausible candidate gene was defined by the following criteria: (a) the gene had a function known to be related to the trait evaluated based on gene ontology term descriptions in Phytozome; (b) BLASTX searches from the Arabidopsis genome returned orthologous protein sequences with functions associated to the phenotypes of interest.

## 5. Conclusions

We performed GWAS using a set of 771,914 SNPs, identifying seven and four QTL for MC and HC, respectively. Above all, chromosome 3 encompassed three QTL harboring promising candidate genes for MC. Three of the QTL associated with HC contained plausible candidate genes related to seed coat and anthocyanin biosynthesis. These favorable QTL alleles will assist the design of market specific flaxseed cultivars with reduced HC while maintaining high MC for food and low MC for feed. The application of the identified SNP markers in molecular-assisted breeding for MC and HC, two complex traits whose phenotyping is labor-intensive and time-consuming, might enable a rapid transfer of favorable alleles into well adapted elite flaxseed cultivars, thus shortening the breeding cycle. Based on our results and previous gene expression studies, we hypothesize that the genetic control of mucilage and hull content in flax might share conserved pathways with Arabidopsis. Further validation of candidate genes, like *LuTT8*, *LuSBT1.7*, *LuMUM4*, and *LuAGL62*, through gene expression analysis or gene editing, will be necessary to validate the hypothesis mentioned above. Characterization of genes underlying the QTL will expand knowledge of the high complexity of cell wall dynamics involved in seed mucilage and seed coat biosynthesis in flaxseed.

## Figures and Tables

**Table 1:** Genome-wide significant peak SNPs for mucilage content (MC) and hull content (HC).

Trait	Marker	Chromosome	Allele	MAF ^1^	−log_10_ (*P*)		*R*^2^ (%)	
					CAR2014	HU2015	CAR2014	HU2015
MC	Lu2-22298066	2	T/C	0.07	8.69	3.41ns ^2^	17.32	ns ^2^
	Lu3-25559600	3	G/T	0.06	7.45	4.13ns ^2^	13.42	ns ^2^
	Lu3-26033342	3	C/G	0.07	7.68	4.23ns ^2^	13.25	ns ^2^
	Lu3-7398487	3	C/T	0.41	4.96ns ^2^	7.02	ns ^2^	11.82
	Lu5-3808878	5	G/A	0.10	8.03	10.21	14.97	16.52
	Lu7-13225294	7	G/A	0.34	8.10	6.91	16.46	12.05
	Lu11-2498303	11	C/G	0.16	7.05	7.47	14.25	13.18
HC	Lu7-6577527	7	A/C	0.13	6.90	7.36	14.66	15.79
	Lu10-21552161	10	G/A	0.09	6.90	6.16ns ^2^	16.32	ns ^2^
	Lu12-5267706	12	C/T	0.06	5.91ns ^2^	6.92	ns ^2^	13.83
	Lu13-2803224	13	T/C	0.06	7.83	8.45	17.43	18.20

^1^ MAF: minor allele frequency; ^2^ ns: not significant at the threshold value –log_10_ (P) = 6.88.

**Table 2:** Candidate genes within LD blocks harboring peak SNPs associated with MC and HC.

Trait	Marker	Gene ID	Scaffold	*A. Thaliana* Ortholog	Gene Bank	Identity (%)	E-Value	Distance from Peak SNP (kb)
MC	Lu3-25559600	Lus10009311	318	*GATL5*	at1g02720	27	7 × 10^−27^	64.41
		Lus10009288	318	*MUM4*	at1g53500	26	4 × 10^−23^	67.02
		Lus10009287	318	*PME36*	at3g60730	61	2 × 10^−110^	70.33
		Lus10009313	318	*SBT1.7*	at5g67360	45	0.0	75.66
	Lu3-26033342	Lus10007101	772	*TT8*	at4g09820	38	5 × 10^−15^	58.92
		Lus10007083	772	*SBT1.7*	at5g67360	39	1 × 10^−152^	49.60
	Lu5-3808878	Lus10008285	489	*NARS1*	at3g15510	52	9 × 10^−45^	100.78
HC	Lu7-6577527	Lus10035456	151	*AGL62*	at5g60440	43	6 × 10^−39^	11.40
	Lu12-5267706	Lus10018306	163	*GH17*	at2g39640	34	9 × 10^−86^	39.93
	Lu13-2803224	Lus10026902	651	*DBR1*	at4g31770	68	0.0	96.87
		Lus10026926	651	*UGT79B1*	at5g54060	25	2 × 10^−32^	238.19

## Supplementary Materials

Supplementary materials can be found at http://www.mdpi.com/1422-0067/19/10/2870/s1.

## Abbreviations

MC	mucilage content
HC	gull content
GWAS	genome-wide association study
LD	linkage disequilibrium
kb	kilobase
SNP	single nucleotide polymorphism
SSR	simple sequence repeat
CAR2014	Vilcún 2014
HU2015	Huichahue 2015
REML	restricted maximum likelihood
AIC	Akaike information criterion
BIC	Bayesian information criterion
BLUE	best linear unbiased estimation
GLM	general linear model
MLM	mixed linear model
PCA	principal component analysis
Q–Q	quantile–quantile

## References

[1] Rabetafika H.N., van Remoortel V., Danthine S., Paquot M., Beckler C. (2011). Flaxseed proteins: Food uses and health benefits. Int. J. Food Sci. Technol..

[2] Kristensen M., Jensen M.G., Aarestrup J., Petersen K., Søndergaard L., Mikkelsen M.S., Astrup A. (2012). Flaxseed dietary fibers lower cholesterol and increase fecal fat excretion, but magnitude of effect depend on food type. Nutr. Metab..

[3] Hunt K., Jones J.K.N. (1962). The structure of linseed mucilage: Part II. Can. J. Chem..

[4] Kaewmanee T., Bagnasco L., Benjakul S., Lanteri S., Morelli C.F., Speranza G., Cosulich M.E. (2014). Characterisation of mucilages extracted from seven Italian cultivars of flax. Food Chem..

[5] Haughn G., Chaudhury A. (2005). Genetic analysis of seed coat development in Arabidopsis. Trends Plant Sci..

[6] Bhatty R.S., Cherdkiatgumchai P. (1990). Compositional analysis of laboratory-prepared and commercial samples of linseed meal and of hull isolated from flax. J. Am. Oil Chem. Soc..

[7] Gajardo H.A., Quian R., Soto-Cerda B. (2017). Agronomic and quality assessment of linseed advanced breeding lines varying in seed mucilage content and their use for food and feed. Crop Sci..

[8] Cherian G., Quezada N. (2016). Egg quality, fatty acid composition and immunoglobulin Y content in eggs from laying hens fed full fat camelina or flax seed. J. Anim. Sci. Biotechnol..

[9] Oomah B.D., Mazza G. (1993). Processing of flaxseed meal: Effect of solvent extraction on physicochemical characteristics. LWT-Food Sci. Technol..

[10] Sosulski F.W., Bakal A. (1969). Isolated proteins from rapeseed, flax and sunflower meals. Can. Inst. Food Sci. Technol. J..

[11] Kessler R.W., Kohler R. (1996). New strategies for exploiting flax and hemp. Chemtech.

[12] Sosulski F., Zadernowski R. (1981). Fractionation of rapeseed meal into hour and hull components. J. Am. Oil Chem. Soc..

[13] Daun J.K., DeClercq D.R. (1988). Quality of yellow and dark seeds in *Brassica campentris* canola varieties Candle and Tobin. J. Am. Oil Chem. Soc..

[14] Francoz E., Ranocha P., Burlat V., Dunand C. (2015). Arabidopsis seed mucilage secretory cells: Regulation and dynamics. Trends Plant Sci..

[15] Venglat P., Xiang D., Qiu S., Stone S.L., Tibiche C., Cram D., Alting-Mees M., Nowak J., Cloutier S., Deyholos M. (2011). Gene expression analysis of flax seed development. BMC Plant Biol..

[16] Oomah B.D., Kenaschuk E.O., Cui W., Mazza G. (1995). Variation in the composition of water-soluble polysaccharides in flaxseed. J. Agric. Food Chem..

[17] Oomah B.D., Mazza G. (1997). Effect of dehulling on chemical composition and physical properties of flaxseed. LWT-Food Sci. Technol..

[18] Diederichsen A., Raney J.P., Duguid S.D. (2006). Variation of mucilage in flax seed and its relationship with other seed characters. Crop Sci..

[19] Spielmeyer W., Green A.G., Bittisnish D., Mendham N., Lagudah E.S. (1998). Identification of quantitative trait loci contributing to Fusarium wilt resistance on an AFLP linkage map of flax (*Linum usitatissimum*). Theor. Appl. Genet..

[20] Asgarinia P., Cloutier S., Duguid S., Rashid K., Mirlohi A.F., Banik M., Saeidi G. (2013). Mapping quantitative trait loci for powdery mildew resistance in flax (*Linum usitatissimum* L.). Crop Sci..

[21] Cloutier S., Ragupathy R., Niu Z., Duguid S. (2011). SSR-based linkage map of flax (*Linum usitatissimum* L.) and mapping of QTL underlying fatty acid composition traits. Mol. Breed..

[22] Kumar S., You F.M., Duguid S., Booker H., Rowland G., Cloutier S. (2015). QTL for fatty acid composition and yield in linseed (*Linum usitatissimum* L.). Theor. Appl. Genet..

[23] Sudarshan G.P., Kulkarni M., Akhov L., Ashe P., Shaterian H., Cloutier S., Rowland G., Wei Y., Selvaraj G. (2017). QTL mapping and molecular characterization of the classical *D* locus controlling seed and flower color in *Linum usitatissimum* (flax). Sci. Rep..

[24] Soto-Cerda B.J., Duguid S., Booker H., Rowland G., Diederichsen A., Cloutier S. (2014). Genomic regions underlying agronomic traits in linseed (*Linum usitatissimum* L.) as revealed by association mapping. J. Integr. Plant Biol..

[25] Soto-Cerda B.J., Duguid S., Booker H., Rowland G., Diederichsen A., Cloutier S. (2014). Association mapping of seed quality traits using the Canadian flax (*Linum usitatissimum* L.) core collection. Theor. Appl. Genet..

[26] Xie D., Dai Z., Yang Z., Sun J., Zhao D., Yang X., Zhang L., Tang Q., Su J. (2018). Genome-wide association study identifying candidate genes influencing important agronomic traits of flax (*Linum usitatissimum* L.) using SLAF-seq. Front. Plant Sci..

[27] You F.M., Xiao J., Li P., Yao Z., Jia G., He L., Kumar S., Soto-Cerda B., Duguid S.D., Booker H.M. (2018). Genome-Wide Association Study and Selection Signatures Detect Genomic Regions Associated with Seed Yield and Oil Quality in Flax. Int. J. Mol. Sci..

[28] Ersoz E.S., Yu J., Buckler E.S., Kriz A., Larkins B. (2009). Applications of linkage disequilibrium and association mapping in maize. Molecular Genetic Approaches to Maize Improvement.

[29] Cloutier S., Niu Z., Datla R., Duguid S. (2009). Development and analysis of EST-SSRs for flax (*Linum usitatissimum* L.). Theor. Appl. Genet..

[30] Cloutier S., Miranda E., Ward K., Radovanovic N., Reimer E., Walichnowski A., Datla R., Rowland G., Duguid S., Ragupathy R. (2012). Simple sequence repeat marker development from bacterial artificial chromosome end sequences and expressed sequence tags of flax (*Linum usitatissimum* L.). Theor. Appl. Genet..

[31] Cloutier S., Ragupathy R., Miranda E., Radovanovic N., Reimer E., Walichnowski A., Ward K., Rowland G., Duguid S., Banik M. (2012). Integrated consensus genetic and physical maps of flax (*Linum usitatissimum* L.). Theor. Appl. Genet..

[32] Ragupathy R., Rathinavelu R., Cloutier S. (2011). Physical mapping and BAC-end sequence analysis provide initial insights into the flax (*Linum usitatissimum* L.) genome. BMC Genom..

[33] Altunkaya A. (2006). Dermal Lubricant and Moisturizer. WO.

[34] Anttila M., Kankaanpää-Anttila B., Sepponen M., Timonen H., Autio K. (2008). Improving of Texture of Dairy Products. WO.

[35] Kracht W., Dänicke S., Kluge H., Keller K., Matzke W., Henning U., Schumann W. (2004). Effect of dehulling of rapeseed on feed value and nutrient digestibility of rape products in pigs. Arch. Anim. Nutr..

[36] Oomah B.D., Mazza G. (1998). Fractionation of flaxseed with a batch dehuller. Ind. Crop Prod..

[37] Yan X.Y., Li J.N., Fu F.Y., Jin M.Y., Chen L., Liu L.Z. (2009). Co-location of seed oil content, seed hull content and seed coat color QTL in three different environments in *Brassica napus* L.. Euphytica.

[38] Saedi G., Rowland G.G. (1999). Seed color and linolenic acid effects on agronomic traits in flax. Can. J. Plant Sci..

[39] Zhao K., Tung C.W., Eizenga G.C., Wright M.H., Ali M.L., Price A.H., Norton G.J., Islam M.R., Reynolds A., Mezey J. (2011). Genome-wide association mapping reveals a rich genetic architecture of complex traits in *Oryza sativa*. Nat. Commun..

[40] Smỳkal P., Bačová-Kerteszová N., Kalendar R., Corander J., Schulman A.H., Pavelek M. (2011). Genetic diversity of cultivated flax (*Linum usitatissimum* L.) germplasm assessed by retrotransposon-based markers. Theor. Appl. Genet..

[41] Soto-Cerda B.J., Diederichsen A., Ragupathy R., Cloutier S. (2013). Genetic characterization of a core collection of flax (*Linum usitatissimum* L.) suitable for association mapping studies and evidence of divergent selection between fiber and linseed types. BMC Plant Biol..

[42] Chandrawati N.S., Kumar R., Kumar S., Singh P.K., Yadav V.K., Ranade S.A., Yadav H.K. (2017). Genetic diversity, population structure and association analysis in linseed (*Linum usitatissimum* L.). Physiol. Mol. Biol. Plants.

[43] Abdurakhmonov I., Abdukarimov A. (2008). Application of association mapping to understanding the genetic diversity of plant germplasm resources. Int. J. Plant Genom..

[44] Xu J., Ranc N., Munos S., Rolland S., Bouchet J.P., Desplant N., Le Paslier M.C., Liang Y., Brunel D., Causse M. (2013). Phenotypic diversity and association mapping for fruit quality traits in cultivated tomato and related species. Theor. Appl. Genet..

[45] Jung M., Ching A., Bhattramakki D., Dolan M., Tingey S., Morgante M., Rafalski A. (2004). Linkage disequilibrium and sequence diversity in a 500-kbp region around the adh1 locus in elite maize germplasm. Theor. Appl. Genet..

[46] Hatzig S.V., Frisch M., Breuer F., Nesi N., Ducournau S., Wagner M.H., Leckband G., Abbadi A., Snowdon R.J. (2015). Genome-wide association mapping unravels the genetic control of seed germination and vigor in *Brassica napus*. Front. Plant Sci..

[47] Pritchard J.K., Stephens M., Rosenberg N.A., Donnelly P. (2000). Association mapping in structured populations. Am. J. Hum. Genet..

[48] Price A.L., Patterson N.J., Plenge R.M., Weinblatt M.E., Shadick N.A., Reich D. (2006). Principal components analysis corrects for stratification in genome-wide association studies. Nat. Genet..

[49] Yu J., Pressoir G., Briggs W., Vroh B., Yamasaki M., Doebley J., McMullen M.D., Gaut B.S., Nielsen D.M., Holland J.B. (2006). A unified mixed-model method for association mapping that accounts for multiple levels of relatedness. Nat. Genet..

[50] Western T.L., Young D.S., Dean G.H., Tan W.L., Samuels A.L., Haughn G.W. (2004). MUCILAGE-MODIFIED4 encondes a putative pectin biosynthetic enzyme developmentally regulated by APETALA2, TRANSPARENT TESTA GLABRA1, and GLABRA2 in the *Arabidopsis* seed coat. Plant Physiol..

[51] Kong Y., Zhou G., Abdeen A.A., Schafhauser J., Richardson B., Atmodjo M.A., Jung J., Wicker L., Mohnen D., Western T., Hahn M.G. (2013). GALACTURONOSYLTRANSFERASE-LIKE5 is involved in the production of *Arabidopsis* seed coat mucilage. Plant Physiol..

[52] Rautengarten C., Usadel B., Neumetzler L., Hartmann J., Büssis D., Altmann T. (2008). A subtilisin-like serine protease essential for mucilage release from Arabidopsis seed coats. Plant J..

[53] Saez-Aguayo S., Ralet M.C., Berger A., Botran L., Ropartz D., Marion-Poll A., North H.M. (2013). PECTIN METHYLESTERASE INHIBITOR6 promotes Arabidopsis mucilage release by limiting methylesterification of homogalacturonan in seed coat epidermal cells. Plant Cell.

[54] Louvet R., Cavel E., Gutierrez L., Guénin S., Roger D., Gillet F., Guerineau F., Pelloux J. (2006). Comprehensive expression profiling of the pectin methylesterase gene family during silique development in *Arabidopsis thaliana*. Planta.

[55] Shi L., Katavic V., Yu Y., Kunst L., Haughn G. (2012). Arabidopsis glabra2 mutant seeds deficient in mucilage biosynthesis produce more oil. Plant J..

[56] Wang R., Li J.N., Chen L., Tang Z.L., Zhang X.K. (2003). Genetic correlation analysis for main characters in yellow-seeded rapeseed lines (*Brassica napus* L.). Chin. J. Oil Crop Sci..

[57] Khan N.A., Booker H., Yu P. (2014). Molecular structures and metabolic characteristics of protein in brown and yellow flaxseed with altered nutrient traits. J. Agri. Food Chem..

[58] Qu C., Hasan M., Lu K., Liu L., Zhang K., Fu F., Wang M., Liu S., Bu H., Wang R. (2015). Identification of QTL for seed coat colour and oil content in *Brassica napus* by association mapping using SSR markers. Can. J. Plant Sci..

[59] Badani A.G., Snowdon R.J., Wittkop B., Lipsa F.D., Baetzel R., Horn R., De Haro A., Font R., Lühs W., Friedt W. (2006). Colocalization of a partially dominant gene for yellow seed colour with a major QTL influencing acid detergent fibre (ADF) content in different crosses of oilseed rape (*Brassica napus*). Genome.

[60] Zhang D., Sun L., Li S., Wang W., Ding Y., Swarm S.A., Li L., Wang X., Tang X., Zhang Z. (2018). Elevation of soybean seed oil content through selection for seed coat shininess. Nat. Plants.

[61] Roszak P., Köhler C. (2011). Polycomb group proteins are required to couple seed coat initiation to fertilization. Proc. Natl. Acad. Sci. USA.

[62] Mizzotti C., Ezquer I., Paolo D., Rueda-Romero P., Guerra R.F., Battaglia R., Rogachev I., Aharoni A., Kater M.M., Caporali E. (2014). SEEDSTICK is a master regulator of development and metabolism in the Arabidopsis seed coat. PLoS Genet..

[63] Marinova K., Pourcel L., Weder B., Schwarz M., Barron D., Routaboul J.M., Debeaujon I., Klein M. (2007). The Arabidopsis MATE transporter TT12 acts as a vacuolar flavonoid/H^+^-antiporter active in proanthocyanidin-accumulating cells of the seed coat. Plant Cell.

[64] Kovinich N., Saleem A., Arnason J.T., Miki B. (2010). Functional characterization of a UDP-glucose:flavonoid 3-O-glucosyltransferase from the seed coat of black soybean (*Glycine max* (L.) Merr.). Phytochemistry.

[65] Xu W., Dubos C., Lepiniec L. (2015). Transcriptional control of flavonoid biosynthesis by MYB–bHLH–WDR complexes. Trends Plant Sci..

[66] Mole S., Waterman P.G. (1986). Tannic acid and proteolytic enzymes: Enzyme inhibition or substrate deprivation?. Phytochemistry.

[67] Diederichsen A., Kusters P.M., Kessler D., Bainas Z., Gugel R.K. (2013). Assembling a core collection from the flax world collection maintained by Plant Gene Resources of Canada. Genet. Resour. Crop Evol..

[68] VSN International (2015). Genstat for Windows.

[69] Korkmaz S., Goksuluk D., Zararsiz G. (2014). MVN: An R Package for Assessing Multivariate Normality. R J..

[70] Bradbury P.J., Zhang Z., Kroon D.E., Casstevens T.M., Ramdoss Y., Buckler E.S. (2007). TASSEL: Software for association mapping of complex traits in diverse samples. Bioinformatics.

[71] Wang Z., Hobson N., Galindo L., Zhu S., Shi D., McDill J., Yang L., Hawkins S., Neutelings G., Datla R. (2012). The genome of flax (*Linum usitatissimum*) assembled de novo from short shotgun sequence reads. Plant J..

[72] You F.M., Xiao J., Li P., Yao Z., Jia G., He L., Zhu T., Luo M.C., Wang X., Deyholos M.K. (2018). Chromosome-scale pseudomolecules refined by optical, physical and genetic maps in flax. Plant J..

[73] Li H., Durbin R. (2009). Fast and accurate short read alignment with Burrows-Wheeler Transform. Bioinformatics.

[74] Li H., Handsaker B., Wysoker A., Fennell T., Ruan J., Homer N., Marth G., Abecasis G., Durbin R. (2009). The sequence alignment/map format and SAMtools. Bioinformatics.

[75] Kumar S., You F.M., Cloutier S. (2012). Genome wide SNP discovery in flax through next generation sequencing of reduced representation libraries. BMC Genom..

[76] You F.M., Deal K.R., Wang J., Britton M.T., Fass J.N., Lin D., Dandekar A.M., Leslie C.A., Aradhya M., Luo M.C. (2012). Genome-wide SNP discovery in walnut with an AGSNP pipeline updated for SNP discovery in allogamous organisms. BMC Genom..

[77] Evanno G., Regnaut S., Goudet J. (2005). Detecting the number of clusters of individuals using the software STRUCTURE: A simulation study. Mol. Ecol..

[78] Francis M.R. (2017). POPHELPER: An R package and web app to analyse and visualise population structure. Mol. Ecol. Resour..

[79] Hill W.G., Weir B.S. (1998). Variances and covariances of squared linkage disequilibria in finite populations. Theor. Popul. Biol..

[80] Turner S.D. (2014). QQMAN: An R package for visualizing GWAS results using Q-Q and manhattan plots. biorXiv.

