# Supplementary material for: Genome-Wide Association Analysis of Mucilage and Hull Content in Flax (Linum usitatissimum L.) Seeds
Source: Int J Mol Sci. 2018 Sep 21;19(10):2870. doi: 10.3390/ijms19102870 (PMC6213135; doi:10.3390/ijms19102870)
Supplement: Supplementary file 1 [file ijms-19-02870-s001.pdf]

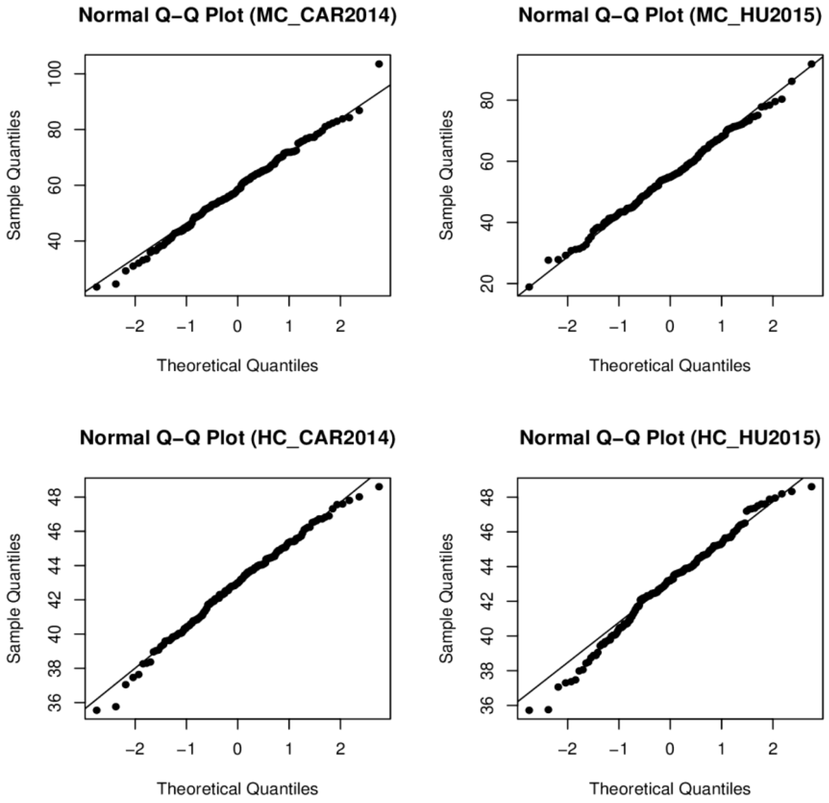

Figure S1. Normal quantile-quantile (Q-Q) plots of phenotypic data for mucilage content (MC) and hull content (HC) across two environments. CAR2014 = Vilcún location 2014, HU2015= Huichahue location 2015.

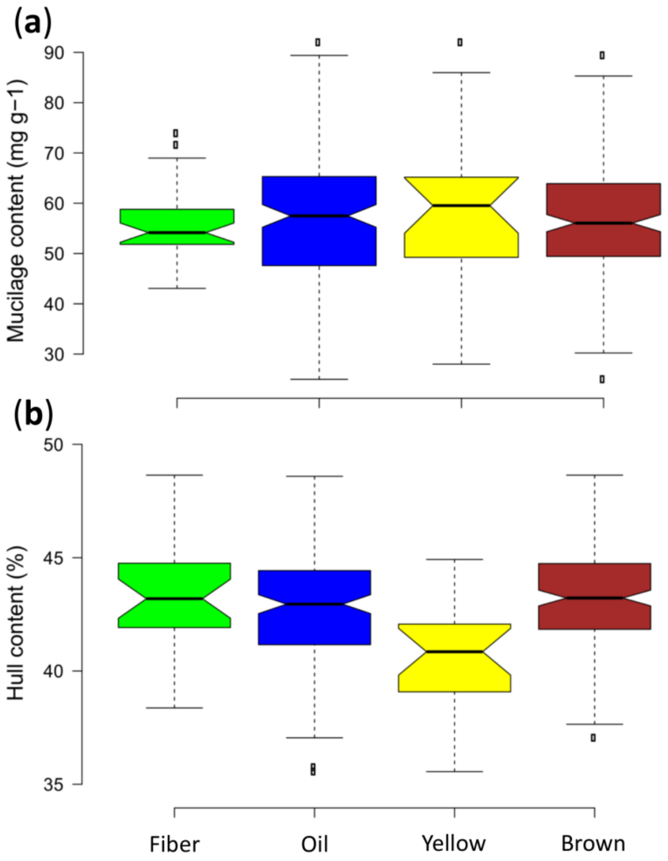

Figure S2. Box plots of phenotypic differences between flax morphotypes (fiber and oil) and seed coat color (yellow and brown). (**a**) mucilage content. (**b**) hull content.

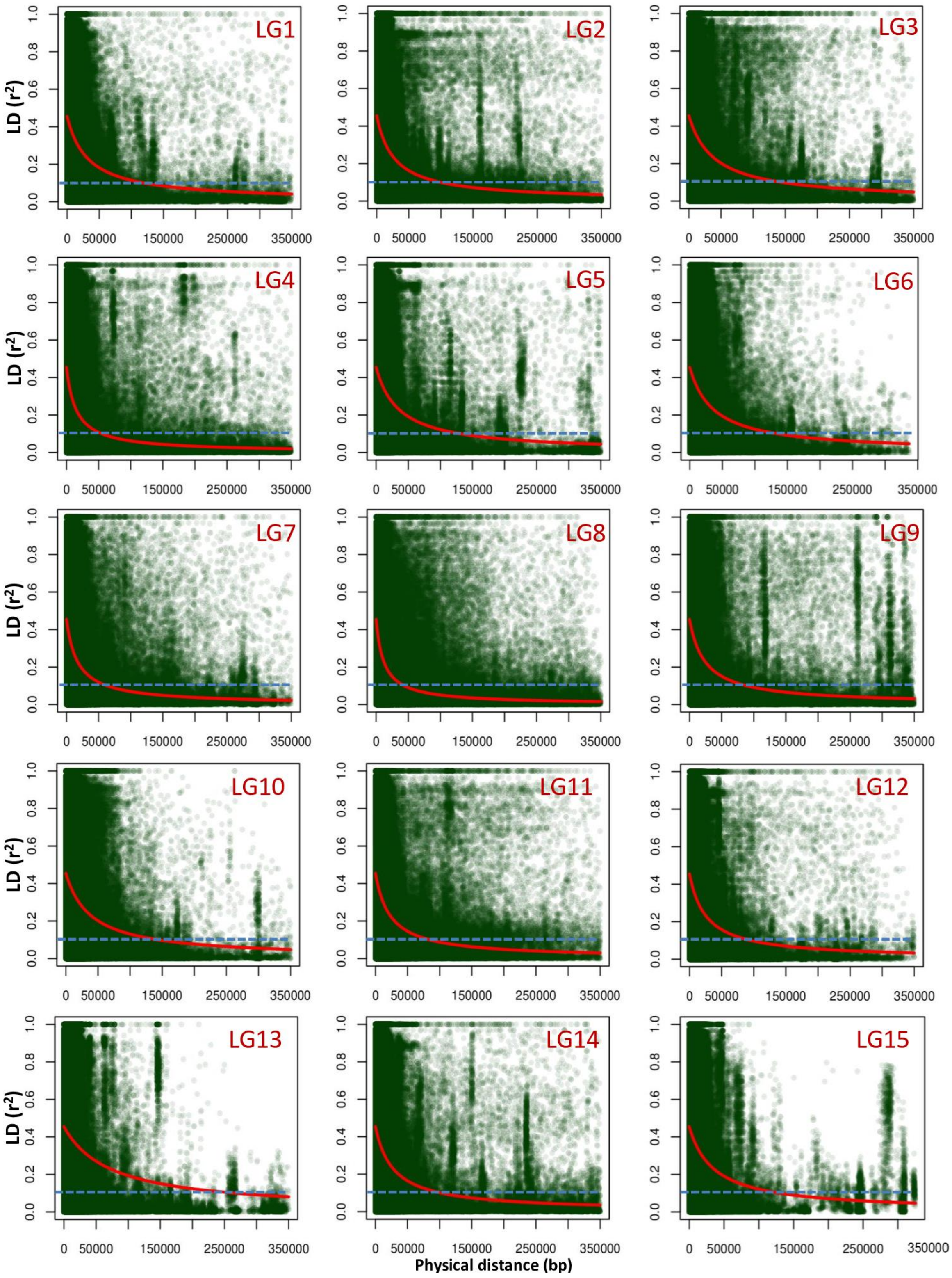

Figure S3. Linkage disequilibrium (LD) decay of r^2 values (red line), against physical distance (bp) across 15 chromosomes of *Linum ussitatissum*. Dashed blue line indicates the cutoff value (r^2 = 0.1) used to determine LD blocks.

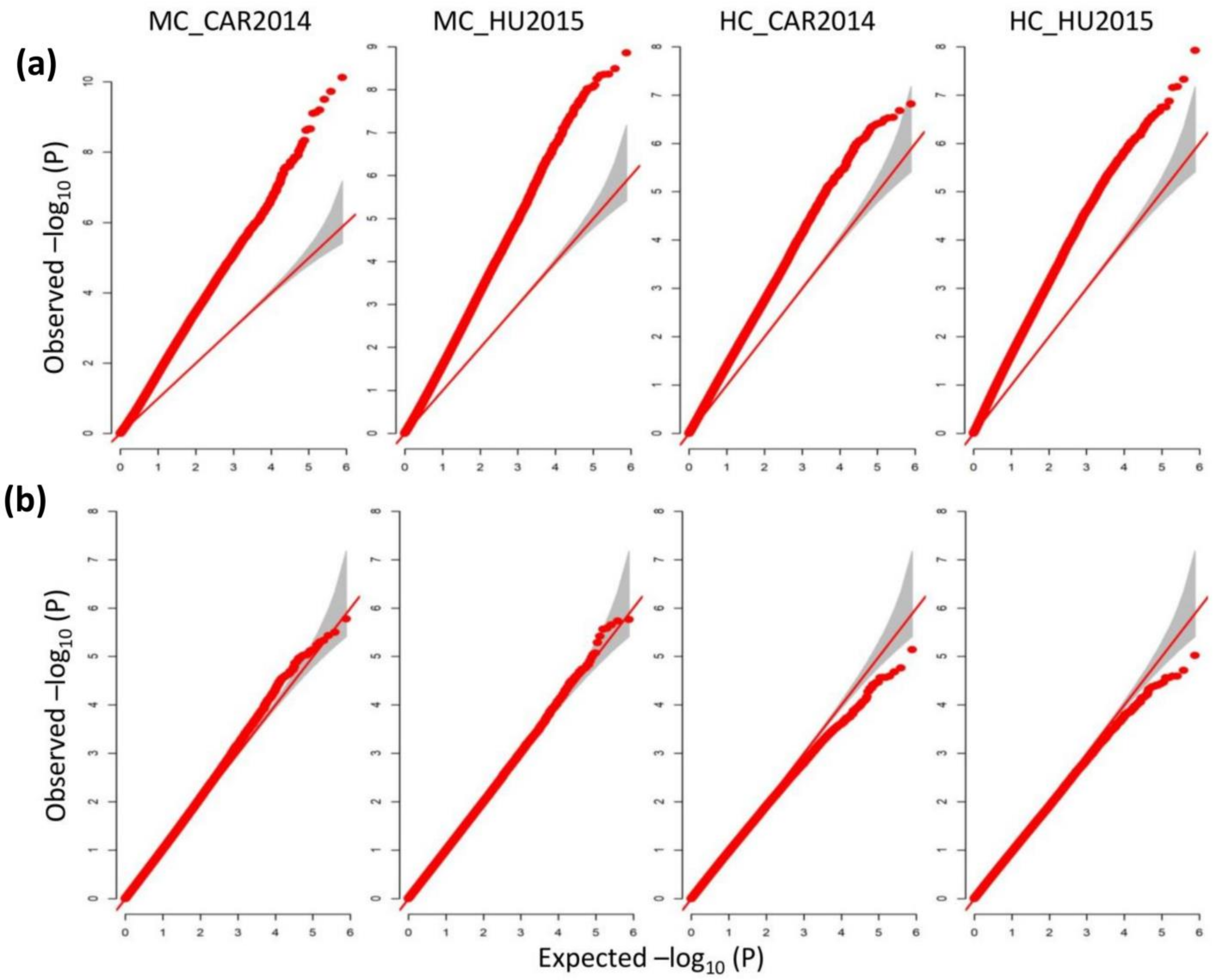

Figure S4. Quantile-quantile (Q-Q) plots of observed P values (y-axis) plotted against expected P values (x-axis) for mucilage content (MC) and hull content (HC) across two environments (CAR2014 = Vilcún location 2014, HU2015 = Huichahue location 2015). (**a**) General linear model (GLM) using the Q matrix as covariate for controlling population structure and cryptic relatedness. (**b**) Mixed linear model (MLM) using the kinship matrix as covariate for controlling population structure and cryptic relatedness.

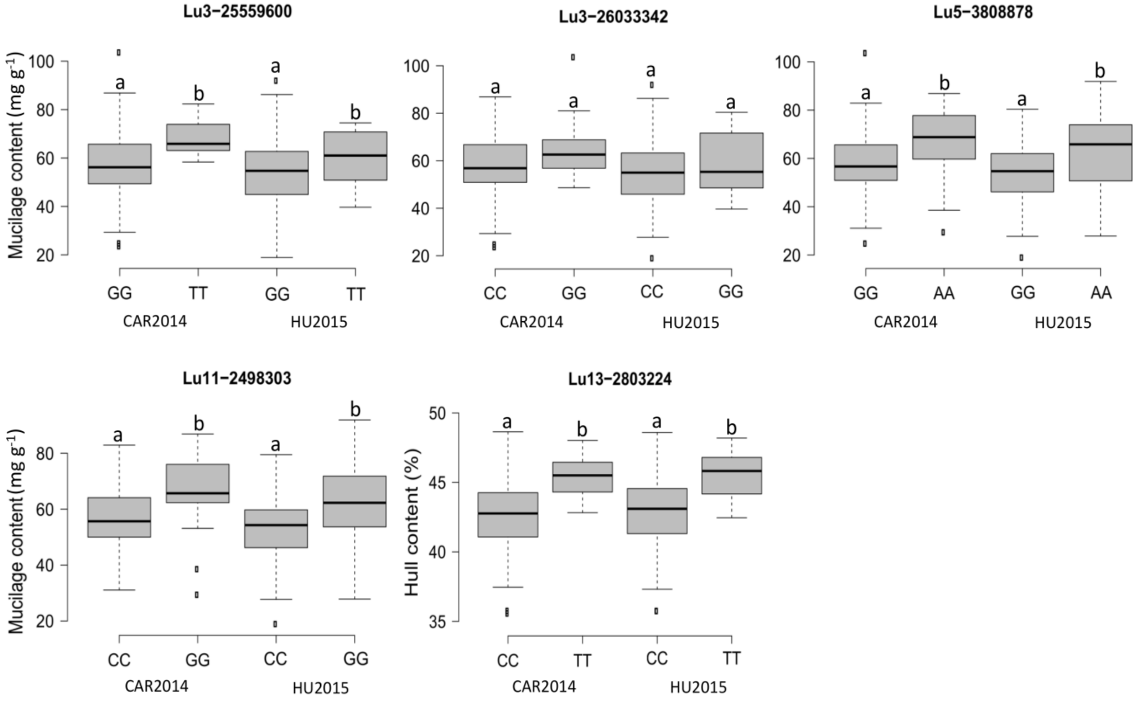

Figure S5. Box plots illustrating the phenotypic differences between flaxseed accessions carrying different alleles of the SNPs associated with mucilage content and hull content across two environments. CAR2014 = Vilcún location 2014, HU2015 = Huichahue location 2015. Different letters indicate significant statistical differences according to the Kruskal-Wallis non-parametric test ($P < 0.05$).

Table S1. Descriptive statistics and normality test for mucilage content and hull content.

Trait	Mean	Std. Dev	Median	Min	Max	Skew	Statistic	P	Normality
MC_CAR2014	58.67	13.33	58.46	23.52	103.57	-0.013	0.9951	0.854	Yes
MC_HU2015	55.04	12.56	54.89	18.88	91.90	0.008	0.9972	0.989	Yes
HC_CAR2014	42.89	2.45	42.98	35.56	48.59	-0.300	0.9920	0.462	Yes
HC_HU2015	43.01	2.57	43.21	35.73	48.59	-0.338	0.9858	0.082	Yes

MC_2014 and MC_2015 are the mucilage contents observed in Vilcún location 2014 and Huichahue location 2015. HC_2014 and HC2015 are the hull contents observed in Vilcún location 2014 and Huichahue location 2015.

Table S2. Peak SNPs and candidate genes for mucilage content and hull content.

Trait	Chr.	SNP	Gene ID	Encoded Protein
MC	2	Lu2-22298066	Lus10014559	Oleosin 21.2 kda-related
			Lus10014558	Compass component swd2
			Lus10014557	Myb-like dna-binding protein myb
			Lus10014555	NADH:ubiquinone reductase
			Lus10014554	tRNA (adenine57-n1/adenine58-n1)-methyltransferase
			Lus10014553	rna helicase
			Lus10014552	DNA cytosine-5- -methyltransferase 3-related
			Lus10014551	ATP-dependent rna helicase ddx1
			Lus10014550	Acid phosphatase/vanadium-dependent haloperoxidase-related protein
			Lus10014549	Protein of unknown function (duf1421)
			Lus10014548	RNA-binding protein 26 (rbm26)
			Lus10014547	RNA-binding protein 26 (rbm26)
			Lus10014546	PWWP domain-containing protein
			Lus10014545	Adenine nucleotide alpha hydrolases-like superfamily protein-related
			Lus10014543	proteasome assembly chaperone 3
			Lus10014542	Serine/threonine-protein kinase osr1/stk39
			Lus10014541	Protein of unknown function (duf1645)
			Lus10014560	Denticleless (dtl, cdt2, dcaf2)
			Lus10014561	Gglycosylphosphatidylinositol transamidase
			Lus10014562	RNA-binding protein-related
			Lus10014563	Phenylalanine--trna ligase alpha subunit
			Lus10014564	GPI inositol-deacylase
			Lus10014565	Peptidyl-prolyl isomerase e
			Lus10014567	Mitochondrial transcription termination factor family protein
			Lus10014568	C3H4 type zinc finger protein-related
			Lus10014569	MYB transcription factor
			Lus10014570	Zinc finger protein-related
			Lus10014571	TPX2 (targeting protein for xklp2) protein family
			Lus10014572	Cystinosin
			Lus10014573	Succinate dehydrogenase (ubiquinone) iron-sulfur subunit
			Lus10014574	Cleavage site for pathogenic type iii effector avirulence factor avr
			Lus10014575	Plastocyanin-like domain
			Lus10014576	15-cis-phytoene synthase
			Lus10014577	Pentatricopeptide repeat repeat-containing protein
			Lus10014578	AGAMOUS-like mads-box protein agl80-related
			Lus10014579	Mitochondrial inner membrane protease atp23
			Lus10014580	Sieve element occlusion n-terminus
			Lus10014581	20s proteasome subunit beta 2
			Lus10014582	Leucine-rich repeat-containing protein
			Lus10014583	Disease resistance protein-related
	3	Lu3-25559600	Lus10009300	Brefeldin A-inhibited guanine nucleotide-exchange protein
			Lus10009301	AT-HOOK motif nuclear localized protein 18-related
			Lus10009302	4-hydroxy-tetrahydrodipicolinate synthase
			Lus10009303	Actin filament-coating protein tropomyosin
			Lus10009305	Kinase A-anchor protein Neurobeachin and related BEACH and WD40 repeat proteins
			Lus10009307	Protein T01H10.8

Table S2. continued.

Trait	Chr.	SNP	Gene ID	Encoded Protein
MC	3	Lu3-25559600	Lus10009308	Carboxylesterase 8-related
			Lus10009309	Poly A polymerase CID PAP -related
			Lus10009310	Poly A polymerase CID PAP -related
			Lus10009311	POLYGALACTURONATE 4-ALPHA-GALACTURONOSYLTRANSFERASE-related
			Lus10009312	Acyltransferase
			Lus10009313	Subtilase family protein-related
			Lus10009314	Predicted transporter
			Lus10009315	Solute carrier family 35
			Lus10009316	Uncharacterized conserved protein, contains SPRY domain
			Lus10009299	PPR repeat (PPR)
			Lus10009298	LOB domain-containing protein 19-related
			Lus10009296	F13O11.17 PROTEIN
			Lus10009295	Protein cornichon homolog 3-related
			Lus10009294	ATP-dependent DNA helicase 2 subunit 2
			Lus10009293	Syntaxin-binding protein 1
			Lus10009292	BTB/POZ domain
			Lus10009291	Protein S-acyltransferase 13-related
			Lus10009290	PPR repeat
			Lus10009289	2'-phosphotransferase [EC:2.7.1.160]
			Lus10009288	UDP-glucuronate 4-epimerase 4
			Lus10009287	Pectinesterase/pectinesterase inhibitor 36-related
			Lus10009286	Auxin-regulated protein-related
			Lus10009285	Ggolgi SNAP receptor complex member 1
			Lus10009284	Plant protein of unknown function (DUF641)
			Lus10009283	PLATZ transcription factor
			Lus10009282	Hydrophobic seed protein
	3	Lu3-26033342	Lus10007093	Phosphoglycerate mutase-like protein
			Lus10007094	N-(long-chain-acyl)ethanolamine deacylase
			Lus10007095	N-(long-chain-acyl)ethanolamine deacylase
			Lus10007096	FALZ-related bromodomain-containing proteins
			Lus10007098	ZINC transport protein ZNTB
			Lus10007099	tRNA-nucleotidyltransferase/poly a polymerase family member
			Lus10007100	MADS Box protein
			Lus10007101	Basic helix-loop-helix (BHLH) DNA binding superfamily protein-related
			Lus10007102	Pathogenesis-related protein 1
			Lus10007104	K(+) efflux antiporter 3, chloroplastic
			Lus10007105	Peptide-methionine (R)-S-oxide reductase
			Lus10007106	Macrophage erythroblast attacher-related
			Lus10007108	Protein trichome birefringence-like 12
			Lus10007109	Arabidopsis protein of unknown function (DUF241)
			Lus10007110	Cysteine-rich secretory protein-related
			Lus10007092	Solute carrier family 31 copper transporters
			Lus10007091	Domain of unknown function (DUF4598)
			Lus10007089	Transcription initiation factor iih-related
			Lus10007088	DNA damage-inducible protein 1
			Lus10007087	Striated muscle activator of Rho-dependent signaling-related
			Lus10007086	Coiled-coil-helix-coiled-coil-helix domain containing 2/nur77

Table S2. continued.

Trait	Chr.	SNP	Gene ID	Encoded Protein
MC	3	Lu3-26033342	Lus10007085	Tryptophan aminotransferase-related
			Lus10007084	Methylmalonate-semialdehyde dehydrogenase
			Lus10007083	SUBTILISIN-LIKE SERINE ENDOPEPTIDASE FAMILY PROTEIN-Related
			Lus10007081	Protein of unknown function (DUF295)
			Lus10007079	L-type lectin-domain containing receptor kinase ix.1-related
			Lus10007077	L-type lectin-domain containing receptor kinase ix.1-related
			Lus10007076	L-type lectin-domain containing receptor kinase ix.1-related
			Lus10007075	L-type lectin-domain containing receptor kinase ix.1-related
			Lus10007074	L-type lectin-domain containing receptor kinase ix.1-related
			Lus10007073	L-type lectin-domain containing receptor kinase ix.1-related
			Lus10007071	Magnesium-dependent phosphatase 1
	3	Lu3-7398487	Lus10040675	Delta(4)-3-oxosteroid 5-beta-reductase
			Lus10040676	Pleiotropic drug resistance proteins (PDR1-15)
			Lus10040677	Ammonium transporter
			Lus10040678	Phospholipase C (plcC)
			Lus10040679	D-isomer specific 2-hydroxyacid dehydrogenase, catalytic domain (2-Hacid_dh)
			Lus10040680	Pentatricopeptide repeat domain (PPR_3)
			Lus10040681	Plekhh protein
			Lus10040682	Soul heme-binding protein
			Lus10040684	MYB domain protein 119
			Lus10040686	Sodium-dependent phosphate transporters
			Lus10040687	Fatty acid omega-hydroxylase (CYP86A1)
			Lus10040689	Thylakoid soluble phosphoprotein TSP9
			Lus10040691	Josephin 1, 2
			Lus10040692	Calcium-dependent lipid-binding domain protein
			Lus10040693	Large subunit ribosomal protein L4
			Lus10040694	Phosphomethylpyrimidine synthase
			Lus10040695	ATP-dependent zinc metalloprotease FTSH 7, chloroplastic-related
			Lus10040696	Duplicated sant dna-binding domain-containing protein
			Lus10040697	LACCASE-16
			Lus10040698	Oligopeptide transporter-related
			Lus10040674	Ca2+:H+ antiporter
			Lus10040670	Nudc domain-containing protein 2
			Lus10040669	Sugar kinase
			Lus10040668	Predicted MutS-related
			Lus10040667	Protein ELF4-like 1
			Lus10040666	Peptidyl-prolyl cis-trans isomerase
			Lus10040665	Glycosyl transferase
			Lus10040664	Phosphatidylinositol phospholipase C, delta
			Lus10040663	serine/threonine-protein kinase-like protein CCR3-related
			Lus10040662	Zinc finger ccch domain-containing protein 66
			Lus10040660	Omega-3 fatty acid desaturase (delta-15 desaturase)
			Lus10040659	WDSAM1 protein
			Lus10040658	Selenoprotein T

Table S2. continued.

Trait	Chr.	SNP	Gene ID	Encoded Protein
MC	3	Lu3-7398487	Lus10040657	NADH:ubiquinone reductase (ndh1)
			Lus10040656	Nuclear movement protein NUDC
			Lus10040654	PHD finger-like domain-containing protein 5A
			Lus10040653	Non-specific serine/threonine protein kinase
	5	Lu5-3808878	Lus10008303	Male germ cell-associated kinase
			Lus10008304	Thaumatin family
			Lus10008305	U3 small nucleolar rna-associated protein 15 homolog
			Lus10008306	Stromal antigen
			Lus10008307	Inositol hexakisphosphate
			Lus10008308	GST-containing FLYWCH ZINC-FINGER protein
			Lus10008309	Cu+-exporting ATPase
			Lus10008310	Protein tyrosine kinase
			Lus10008299	HEAT and armadillo repeat-containing protein
			Lus10008298	Methyltransferase pmt18-related
			Lus10008297	IP01149P-related
			Lus10008295	F-box domain
			Lus10008294	Transmembrane protein induced by tumor necrosis factor alpha
			Lus10008293	Beta-1,3-glucosyltransferase
			Lus10008291	AP2 domain
			Lus10008288	Nuclear pore complex protein Nup98-Nup96
			Lus10008286	Membrane associated ring finger
			Lus10008285	NAC transcription factor-like 9
	7	Lu7-13225294	Lus10007180	partner of Y14 and mago
			Lus10007175	Myb/SANT-like DNA-binding domain
			Lus10007174	HSA (HSA)
			Lus10007173	PROTEIN PHOTOPERIOD-INDEPENDENT EARLY FLOWERING 1
			Lus10007172	DNA helicase
	11	Lu11-2498303	Lus10041945	Stress-induced protein Di19, C-terminal
			Lus10041946	Elongation factor Tu
			Lus10041947	LWNK lysine deficient protein kinase
			Lus10041948	Mitochondrial/chloroplast ribosomal protein L19
			Lus10041950	High-affinity nitrate transporter 3.1-related
			Lus10041951	CYTOCHROME P450 71A12-related
			Lus10041952	Nnucleolin
			Lus10041953	Leucine-rich repeat receptor-like serine/threonine/tyrosine-protein kinase SOBIR1
			Lus10041954	Mitochondrial domain of unknown function (DUF1713)
			Lus10041955	Enhanced disease susceptibility 5-related
			Lus10041956	Glycerol kinase
			Lus10041957	snRNA-activating protein complex subunit 3
			Lus10041958	Kinesin motor family protein
			Lus10041959	Purple acid phosphatase 10
			Lus10041960	Finger and BAH motif containing putative transcription factor-related
			Lus10041961	B3 DNA binding domain
			Lus10041962	Major facilitator superfamily protein
			Lus10041963	Chromatin remodeling protein-related
			Lus10041964	26S proteasome regulatory subunit T4
			Lus10041965	Nudix hydrolase
			Lus10041966	Photosystem I subunit V

Table S2. continued.

Trait	Chr.	SNP	Gene ID	Encoded Protein
MC	11	Lu11-2498303	Lus10041967	Phospholipase A1-IIDELTA
			Lus10041968	Histidinol-phosphatase
			Lus10041969	Dehydrin
			Lus10041970	Zinc finger, C3HC4 type (RING finger)
			Lus10041940	50S ribosomal protein l13, chloroplastic
			Lus10041939	transmembrane EMP24 domain-containing protein
			Lus10041937	Leucine Rich Repeat 1
			Lus10041936	serine/threonine-protein kinase WNK with no lysine -related
			Lus10041935	SBP domain
			Lus10041934	Eukaryotic translation initiation factor SUI1
			Lus10041933	Eukaryotic translation initiation factor SUI1
			Lus10041932	PROTON PUMP-INTERACTOR 1-RELATED
			Lus10041931	Uncharacterized conserved protein
			Lus10041929	VQ motif
			Lus10041928	Methylthioribulose 1-phosphate dehydratase
			Lus10041927	PPR repeat
			Lus10041926	Pollen ole E 1 allergen and extensin family protein
			Lus10041925	Small subunit ribosomal protein S20
			Lus10041924	PROTEIN CUP-SHAPED COTYLEDON 1-related
HC	7	Lu7-6577527	Lus10035457	No annotated protein
			Lus10035458	Probable glycosyltransferase STELLO1
			Lus10035459	Amidophosphoribosyltransferase
			Lus10035460	tRNA-dihydrouridine(47) synthase (NAD(P)(+))
			Lus10035461	No annotated protein
			Lus10035462	Protein basic pentacysteine4-related
			Lus10035456	AGAMOUS-LIKE MADS-BOX PROTEIN AGL62
			Lus10035455	No annotated protein
			Lus10035454	No annotated protein
			Lus10035453	Phosphate-transporting ATPase
			Lus10035452	UDP-GLYCOSYLTRANSFERASE 82A1
			Lus10035451	Galactose-binding domain-like
			Lus10035450	Villin 1
	10	Lu10-21552161	Lus10020237	Leucine-rich repeat-containing protein
			Lus10020238	Disease resistance protein-related
			Lus10020239	Deoxyhypusine synthase
			Lus10020240	Plant protein of unknown function (DUF868)
			Lus10020241	N-MYC downstream regulated
			Lus10020242	Tetratricopeptide repeat protein
			Lus10020243	Pentatricopeptide repeat-containing protein
			Lus10020244	Serine/threonine-protein kinase NEK1
			Lus10020245	PUR-transcriptional activator
			Lus10020246	AD039 HT014 thioredoxin family TRP26
			Lus10020247	GDP-mannose 3,5-epimerase
			Lus10020248	Ammonium transporter
			Lus10020249	Agglutinin domain
			Lus10020250	Chitinase-related
			Lus10020261	Glucan endo-1,3-beta-glucosidase 8-related
			Lus10020236	Serine/threonine-protein kinase wnk1-related
	12	Lu12-5267706	Lus10018295	CPG binding protein
			Lus10018296	SNARE proteins
			Lus10018297	Exocyst complex component EXO84C
			Lus10018298	MEF2B protein

Table S2. continued.

Trait	Chr.	SNP	Gene ID	Encoded Protein
HC	12	Lu12-5267706	Lus10018300	Ribonuclease H2 subunit C
			Lus10018301	Phloem protein 2
			Lus10018302	Polygalacturonase
			Lus10018303	Phloem protein 2
			Lus10018304	Phloem protein 2
			Lus10018305	Myo-inositol-1(or 4)-monophosphatase
			Lus10018306	O-GLYCOSYL HYDROLASES FAMILY 17 protein
			Lus10018307	Kelch motif
			Lus10018308	Disease resistance protein-related
			Lus10018309	Disease resistance protein-related
			Lus10018310	Lecithin-cholesterol acyltransferase-like 4
			Lus10018311	GDP-mannose transporter
			Lus10018314	Exordium like 7
			Lus10018315	LACTOSYLCERAMIDE 4-ALPHA-GALACTOSYLTRANSFERASE
			Lus10018316	DCD (development and cell death) domain protein
			Lus10018317	Domain of unknown function (DUF3475)
			Lus10018318	Proteasome subunit
			Lus10018319	glycine cleavage system H protein
			Lus10018320	Plant protein of unknown function (DUF247)
			Lus10018321	Coatomer subunit delta
			Lus10018294	Inhibitor of apoptosis
			Lus10018293	RBR family ring finger and ibr domain-containing
			Lus10018292	Ring finger domain-containing
			Lus10018291	Peptide-O-fucosyltransferase
			Lus10018290	eukaryotic translation initiation factor 2C
			Lus10018289	regulatory protein NPR1
			Lus10018288	mitotic-spindle organizing protein 1 (MZT1, GIP1, GIP2)
			Lus10018287	CT120 protein
			Lus10018286	Aspartyl protease family protein
			Lus10018285	RAS-related protein Rab-5C
			Lus10018284	Small subunit ribosomal protein SAe
			Lus10018283	Homeodomain-like superfamily protein-related
			Lus10018282	Protein SEY1 homolog
			Lus10018281	PROTEIN ROOT HAIR DEFECTIVE 3 HOMOLOG 2
			Lus10018280	S-linalool synthase
			Lus10018279	F21B23.6 protein
			Lus10018278	PPR repeat
			Lus10018277	Multiple RNA-binding domain-containing protein 1
			Lus10018276	Gamma-glutamylcyclotransferase
	13	Lu13-2803224	Lus10026881	Superoxide dismutase [FE] 2, chloroplastic
			Lus10026882	Exosome complex protein LRP1
			Lus10026883	No annotated protein
			Lus10026884	PPR repeat
			Lus10026885	Protein phosphatase 1G
			Lus10026886	Protein phosphatase 2C
			Lus10026887	Phospholipid hydroperoxide glutathione peroxidase 1, chloroplastic-related
			Lus10026888	PPR repeat family
			Lus10026889	No annotated protein
			Lus10026890	Folate-biopterin transporter 6-related
			Lus10026891	AIG1 domain-containing protein-related

Table S2. continued.

Trait	Chr.	SNP	Gene ID	Encoded Protein
HC	13	Lu13-2803224	Lus10026892	U2-associated protein SR140
			Lus10026893	Folate-biopterin transporter 2-related
			Lus10026894	Mitogen-activated protein kinase kinase kinase 19-related
			Lus10026895	Monogalactosyldiacylglycerol synthase
			Lus10026896	Potential DNA-binding domain (zf-C3Hc3H)
			Lus10026897	VQ motif-containing protein 4-like
			Lus10026898	Polyketide cyclase
			Lus10026899	Protein disulfide-isomerase
			Lus10026900	Spindle and kinetochore-associated protein 2
			Lus10026901	Zinc finger ccch-type with g patch domain-containing protein
			Lus10026902	Lariat debranching enzyme [EC:3.1.-.-]
			Lus10026903	Peroxidase 18-related
			Lus10026904	Translation initiation factor 3 subunit B
			Lus10026905	Aspartyl protease family protein
			Lus10026906	Aspartyl protease family protein
			Lus10026923	Xyloglucan 4-glucosyltransferase
			Lus10026926	Flavonol 3-O-glucosyltransferase
			Lus10026927	Glucosyl/glucuronosyl transferases
			Lus10026880	Early nodulin-like protein 1-related
			Lus10026879	NAC domain containing protein 87
			Lus10026878	Thioredoxin-like 1-1, chloroplastic-related
			Lus10026877	Glutamate receptor 2.5-related
			Lus10026876	Glutamate receptor 2.1-related
			Lus10026875	Pyruvate kinase-related
			Lus10026874	Protein kinase C substrate 80K-H
			Lus10026873	Cupin 1
			Lus10026872	Benzyl alcohol O-benzoyltransferase
			Lus10026867	Ribokinase
			Lus10026865	Fructokinase
			Lus10026863	Endoglucanase 7
			Lus10026862	SCC1 / RAD21 family member
			Lus10026861	Beta-glucosidase
			Lus10026860	Small nuclear ribonucleoprotein F
			Lus10026859	Type 2A phosphatase activator TIP41
			Lus10026849	CALRETICULIN-3

MC: mucilage content; HC: Hull content.

Table S3. Flaxseed accessions used for GWA analysis.

N°	Accession	Seed Color
1	F_AFG_U_CN100952	Brown
2	F_BLR_C_CN101038	Brown
3	F_EGY_C_CN98826	Brown
4	F_FRA_C_CN101392	Brown
5	F_FRA_C_CN97351	Brown
6	F_FRA_L_CN98710	Brown
7	F_JPN_C_CN98072	Brown
8	F_NLD_C_CN18987	Brown
9	F_NLD_C_CN18997	Brown
10	F_NLD_C_CN97424	Yellow
11	F_NLD_U_CN101407	Brown
12	F_ROM_U_CN101405	Brown
13	F_RUS_B_CN101039	Brown
14	F_RUS_B_CN101055	Brown
15	F_RUS_B_CN101114	Brown
16	F_RUS_B_CN101115	Brown
17	F_RUS_B_CN101116	Brown
18	F_RUS_B_CN101127	Brown
19	F_RUS_C_CN101099	Brown
20	F_RUS_C_CN101136	Brown
21	F_RUS_C_CN32542	Brown
22	F_RUS_C_CN35791	Brown
23	F_RUS_L_CN97531	Brown
24	F_RUS_U_CN101348	Brown
25	F_RUS_U_CN101394	Brown
26	F_RUS_U_CN101395	Brown
27	F_RUS_U_CN101402	Brown
28	F_TUR_U_CN101382	Brown
29	F_TUR_U_CN101385	Brown
30	F_TUR_U_CN101386	Brown
31	F_UKR_U_CN101378	Brown
32	F_UKR_U_CN101379	Brown
33	F_USA_B_CN98926	Brown
34	O_AFG_U_CN100807	Brown
35	O_AFG_U_CN101338	Brown
36	O_ARG_B_CN113346	Brown
37	O_ARG_B_CN98037	Brown
38	O_ARG_C_CN97214	Yellow
39	O_ARG_C_CN97953	Brown
40	O_ARG_C_CN97961	Brown
41	O_ARG_C_CN97967	Brown
42	O_ARG_C_CN98007	Brown
43	O_ARG_C_CN98027	Brown
44	O_ARG_C_CN98039	Brown
45	O_ARG_C_CN98279	Brown
46	O_ARG_C_CN98634	Brown
47	O_ARM_U_CN101373	Brown
48	O_AUS_C_CN98984	Brown
49	O_CAN_B_CN101463	Yellow
50	O_CAN_B_CN101472	Brown
51	O_CAN_B_CN101496	Brown

Table S3. continued.

N°	Accession	Seed Color
52	O_CAN_B_CN101554	Yellow
53	O_CAN_B_CN101560	Brown
54	O_CAN_B_CN101565	Yellow
55	O_CAN_B_CN101594	Yellow
56	O_CAN_B_CN101598	Yellow
57	O_CAN_C_CDCBethune	Brown
58	O_CAN_C_CDCGold	Yellow
59	O_CAN_C_CDCMons	Brown
60	O_IND_C_CN98440	Brown
61	O_CAN_C_CDCSorrel	Brown
62	O_CAN_C_CN101413	Brown
63	O_CAN_C_CN18981	Brown
64	O_CAN_C_CN19004	Brown
65	O_CAN_C_CN19005	Brown
66	O_CAN_C_CN19159	Brown
67	O_CAN_C_CN33385	Brown
68	O_CAN_C_CN33386	Brown
69	O_CAN_C_CN33388	Brown
70	O_CAN_C_CN33389	Brown
71	O_CAN_C_CN52732	Brown
72	O_CAN_C_CN97571	Brown
73	O_CAN_C_CN97633	Brown
74	O_CAN_C_CN97671	Brown
75	O_CAN_C_Macbeth	Brown
76	O_CAN_C_PrairieBlue	Brown
77	O_CAN_C_PrairieThunder	Brown
78	O_CAN_C_Shape	Brown
79	O_CAN_C_UGG102-2	Brown
80	O_CSK_C_CN100884	Yellow
81	O_CZE_C_CN100805	Yellow
82	O_CZE_C_CN98683	Brown
83	O_DEU_B_CN113306	Brown
84	O_DEU_B_CN97430	Brown
85	O_DEU_C_CN100881	Brown
86	O_DEU_C_CN97886	Brown
87	O_EGY_U_CN101329	Brown
88	O_ETH_B_CN19007	Brown
89	O_ETH_C_CN96988	Yellow
90	O_ETH_C_CN96991	Brown
91	O_ETH_C_CN96992	Brown
92	O_ETH_C_CN97004	Brown
93	O_FRA_B_CN100863	Yellow
94	O_FRA_C_CN97350	Brown
95	O_FRA_C_CN98712	Brown
96	O_FRA_C_CN98734	Brown
97	O_GBR_C_CN101265	Brown
98	O_GEO_U_CN101366	Brown
99	O_HUN_C_CN100883	Brown
100	O_HUN_C_CN97238	Brown
101	O_HUN_C_CN97287	Brown
102	O_HUN_C_CN97300	Brown

Table S3. continued.

N°	Accession	Seed Color
103	O_HUN_C_CN98263	Brown
104	O_HUN_C_CN98263B	Brown
105	O_HUN_C_CN98275	Brown
106	O_HUN_C_CN98276	Brown
107	O_HUN_C_CN98278	Brown
108	O_HUN_C_CN98854	Brown
109	O_IND_C_CN101208	Yellow
110	O_IND_C_CN97306	Brown
111	O_IND_C_CN98057	Yellow
112	O_IND_C_CN98250	Brown
113	O_IND_C_CN98364	Brown
114	O_IND_C_CN98370	Brown
115	O_IND_C_CN98415	Brown
116	O_IND_C_CN98961	Brown
117	O_IND_C_CN98974	Brown
118	O_IND_L_CN98240	Brown
119	O_IND_U_CN101308	Brown
120	O_IND_U_CN101310	Brown
121	O_IRL_C_CN98192	Brown
122	O_IRN_C_CN97050	Brown
123	O_IRN_C_CN97139	Brown
124	O_IRN_L_CN97129	Brown
125	O_IRN_L_CN97129B	Brown
126	O_JPN_C_CN97470	Brown
127	O_LTU_C_CN101237	Brown
128	O_MAR_B_CN101026	Brown
129	O_NLD_C_CN113300	Brown
130	O_NLD_C_CN18993	Brown
131	O_NLD_C_CN97458	Brown
132	O_NLD_C_CN97613	Brown
133	O_NLD_C_CN98056	Brown
134	O_PAK_C_CN100629	Brown
135	O_PAK_C_CN97056	Brown
136	O_PAK_C_CN97064	Brown
137	O_PAK_C_CN97092	Yellow
138	O_PAK_C_CN97096	Yellow
139	O_PAK_C_CN97103	Brown
140	O_PAK_C_CN98237	Brown
141	O_PAK_C_CN98239	Brown
142	O_PAK_L_CN97083	Brown
143	O_POL_B_CN98733	Brown
144	O_ROM_C_CN100674	Brown
145	O_ROM_C_CN97321	Brown
146	O_RUS_B_CN101241	Brown
147	O_RUS_B_CN101289	Brown
148	O_RUS_B_CN101296	Brown
149	O_RUS_B_CN101301	Brown
150	O_RUS_C_CN96845	Yellow
151	O_RUS_C_CN96846	Brown
152	O_RUS_C_CN97484	Brown
153	O_RUS_C_CN97489	Brown

Table S3. continued.

N°	Accession	Seed Color
154	O_RUS_C_CN97520	Brown
155	O_TUR_U_CN101332	Brown
156	O_UKR_C_CN30860	Brown
157	O_UNK_C_CN100547	Brown
158	O_UNK_C_CN30861	Brown
159	O_URY_C_CN98100	Brown
160	O_USA_B_CN113314	Brown
161	O_USA_B_CN97404	Brown
162	O_USA_B_CN97679	Brown
163	O_USA_B_CN97679B	Brown
164	O_USA_C_CN18994	Brown
165	O_USA_C_CN19160	Yellow
166	O_USA_C_CN33399	Brown
167	O_USA_C_CN33400	Brown
168	O_USA_C_CN33992	Brown
169	O_USA_C_CN97377	Brown
170	O_USA_C_CN97396	Yellow
171	O_USA_C_CN97403	Brown
172	O_USA_C_CN97407	Brown
173	O_USA_C_CN97463	Brown
174	O_USA_C_CN97586	Brown
175	O_USA_C_CN97639	Brown
176	O_USA_C_CN97642	Brown
177	O_USA_C_CN97649	Brown
178	O_USA_C_CN97718	Brown
179	O_USA_C_CN97881	Brown
180	O_USA_C_CN97890	Brown
181	O_USA_C_CN97921	Brown
182	O_USA_C_CN98231	Brown
183	O_USA_C_CN98541	Brown
184	O_USA_C_CN98542	Brown
185	O_USA_C_CN98613	Brown
186	O_USA_C_CN98821	Brown
187	U_ARG_C_CN97341	Brown
188	U_ESP_U_CN101327	Brown
189	U_ETH_L_CN100895B	Brown
190	U_MAR_C_CN98193	Brown
191	U_NZL_B_CN100797	Yellow
192	U_NZL_B_CN100797B	Yellow
193	U_PAK_C_CN100790	Brown
194	U_ROM_C_CN100678	Brown
195	U_RUS_L_CN97483	Brown
196	U_TUR_U_CN100828	Brown
197	U_USA_B_CN97402	Brown
198	U_USA_B_CN97406	Brown
199	U_USA_C_CN97397	Brown
200	U_USA_C_CN97453	Brown

F = Fiber morphotype, O = Oil morphotype, U = Unknown morphotype, C = Cultivar, B = Breeding material, L= Landrace, U = Unknown breeding status. CN = Canadian number at Plant Gene Resources of Canada (PGRC).
